# A Novel RANKL/RANK Inhibitor IMB-R38 Inhibits Osteoporosis Through Regulating Bone Metabolism

**DOI:** 10.3390/ijms262412151

**Published:** 2025-12-17

**Authors:** Yuyan Zhang, Xinwei Wei, Ren Sheng, Guijun Yang, Xiaowan Han, Jingrui Wang, Chao Liu, Shunwang Li, Lijuan Lei, Weilian Jiang, Yang Lun, Shuyi Si, Jing Zhang, Yanni Xu

**Affiliations:** State Key Laboratory of Bioactive Substance and Function of Natural Medicines, NHC Key Laboratory of Microbial Drugs, National Center for New Microbial Drug Screening, Institute of Medicinal Biotechnology, Chinese Academy of Medical Sciences & Peking Union Medical College (CAMS&PUMC), Tiantan Xili 1#, Beijing 100050, China; yuyanzhang1999@163.com (Y.Z.); weixinwei715814@163.com (X.W.); shengren0427@163.com (R.S.); gjyang_5987@163.com (G.Y.); hanxiaowan@imm.ac.cn (X.H.); wangjr0319@163.com (J.W.); 13521199061@163.com (C.L.); lisw973@163.com (S.L.); leilijuanlj@163.com (L.L.); 3292934477wl@gmail.com (W.J.); lunyang_0730@sina.com (Y.L.); sisy@imb.pumc.edu.cn (S.S.)

**Keywords:** osteoporosis, RANKL, RANK, RANKL/RANK inhibitor, osteoclast, osteoblast

## Abstract

Osteoporosis is a systemic skeletal disease that severely impairs the health of the elderly population. The interaction between the receptor activator of the NF-κB ligand (RANKL) and its receptor RANK is critical for osteoclast differentiation and function. Therefore, targeting the RANKL/RANK interaction represents a promising strategy for osteoporosis. In this study, we employed a newly established yeast two-hybrid system based on RANKL/RANK interaction and identified IMB-R38, a novel benzamide compound that dose-dependently blocked RANKL/RANK interaction by inhibiting the growth of AH109 cells harboring pAD-RANKL/pBD-RANK plasmids in quadruple-dropout medium. IMB-R38 significantly suppressed osteoclast differentiation, disrupted F-actin ring formation, and downregulated the expression of osteoclast-specific genes, including NFATc1 and MMP9 in RANKL-induced RAW264.7 macrophages. IMB-R38 also promoted osteoblast differentiation by upregulating the expression of osteogenic genes. Importantly, in a dexamethasone (DXM)-induced osteoporotic zebrafish model, IMB-R38 significantly increased bone mineralization, with anti-osteoporosis efficacy superior to that of alendronate sodium (Alen). RT-qPCR assays showed that IMB-R38 significantly upregulated the mRNA expression of osteogenesis genes (*Bmp2*, *Runx2a*, *Runx2b*, *Sp7*, *Alp*, and *Oc*) while markedly downregulating that of the osteoclastogenesis genes (*Mmp9*, *Mmp13,* and *Mmp2*) compared with the DXM group. Mechanistically, an SPR assay confirmed that IMB-R38 directly binds with RANK but not RANKL to disrupt RANKL/RANK interaction. Furthermore, Asp168 of RANK was identified as a key amino acid that mediates both RANKL interaction and IMB-R38 binding. The inhibition of RANKL/RANK by IMB-R38 suppressed JNK phosphorylation and, consequently, osteoclast differentiation and function. Collectively, our findings identify IMB-R38 as a novel RANKL/RANK inhibitor with therapeutic potential for osteoporosis through its regulation of bone metabolism.

## 1. Introduction

Osteoporosis is a systemic skeletal disorder characterized by low bone mass and deterioration of bone tissue microarchitecture, which increases bone fragility and fracture susceptibility [1,2,3]. As one of the most common metabolic bone diseases, osteoporosis severely impairs the quality of life, particularly among middle-aged and elderly individuals [4,5]. Global estimates indicate that approximately one in three women and one in five men over the age of 50 are likely to experience an osteoporotic fracture [6]. After fractures, the risk of subsequent fractures and mortality rises significantly, imposing a heavy socioeconomic burden on healthcare systems [7]. Bisphosphonates, the most commonly prescribed clinical anti-osteoporosis drugs, function by inhibiting osteoclast (OC) differentiation and activity. However, their clinical use is limited by severe gastrointestinal side effects [8,9]. Estrogen supplements represent another therapeutic option, yet long-term administration increases the risk of cardiovascular disease and cancer [10]. Teriparatide, a parathyroid hormone analog, is restricted to a 24-month duration and is contraindicated in osteoporotic patients with a history of malignancy [11]. Thus, there remains an unmet clinical need for safer and more effective anti-osteoporosis drugs.

Bone is a dynamic organ that undergoes a continuous renewal and remodeling process. Bone remodeling is a cyclic metabolic process mediated by OC-induced bone resorption and osteoblast (OB)-mediated bone formation, maintaining the homeostasis of bone architecture [12,13]. Imbalanced bone remodeling is a key pathogenic factor for the pathogenesis of osteoporosis [12]. In osteoporosis patients, OC-mediated bone resorption exceeds OB-mediated bone formation, leading to bone mass loss and increased fracture risk. The differentiation of mature and functional OCs is initiated by the interaction between the nuclear factor-κB (NF-κB) ligand (RANKL) (synthesized and secreted by OBs) and receptor activator of NF-κB (RANK) (displayed by OCs) [14,15,16]. Upon RANKL binding to RANK, several signaling pathways are simulated including JNK (c-Jun N-terminal kinase), p38 (p38 mitogen-activated protein kinase), PI3K/AKT (Phosphoinositide 3-kinase/Protein kinase B), and MAPK/ERK (mitogen-activated protein kinase/extracellular signal-regulated kinase) [15]. Osteoprotegerin (OPG), secreted by OBs, acts as a decoy receptor that sequesters RANKL, thereby inhibiting RANKL-RANK binding [17]. Therefore, blocking the RANKL/RANK interaction to prevent osteoclastogenesis can relieve osteoporosis. Denosumab, a monoclonal anti-RANKL antibody, has been clinically approved for osteoporosis treatment [18,19]. However, antibody drugs are associated with high expenses and injection administration, highlighting the urgent need to develop small-molecule RANKL/RANK inhibitors.

The yeast two-hybrid system is a powerful tool for studying protein–protein interactions and has been widely utilized in drug discovery [20]. In this study, we successfully developed and validated a yeast two-hybrid system specifically designed to identify inhibitors that block the interaction between RANKL and RANK. Using this system, a novel benzamide compound IMB-R38 was identified as a positive hit. The anti-osteoporotic effect and mechanism of IMB-R38 were then evaluated through both in vitro studies and in two dexamethasone (DXM)-induced osteoporotic zebrafish models. Our results demonstrate that IMB-R38 is a promising lead compound for the development of novel osteoporosis therapeutics, with potentially superior efficacy compared to alendronate sodium (Alen).

## 2. Results

### 2.1. Construction and Validation of RANKL/RANK Interaction Yeast Two-Hybrid System

**Figure 1:**
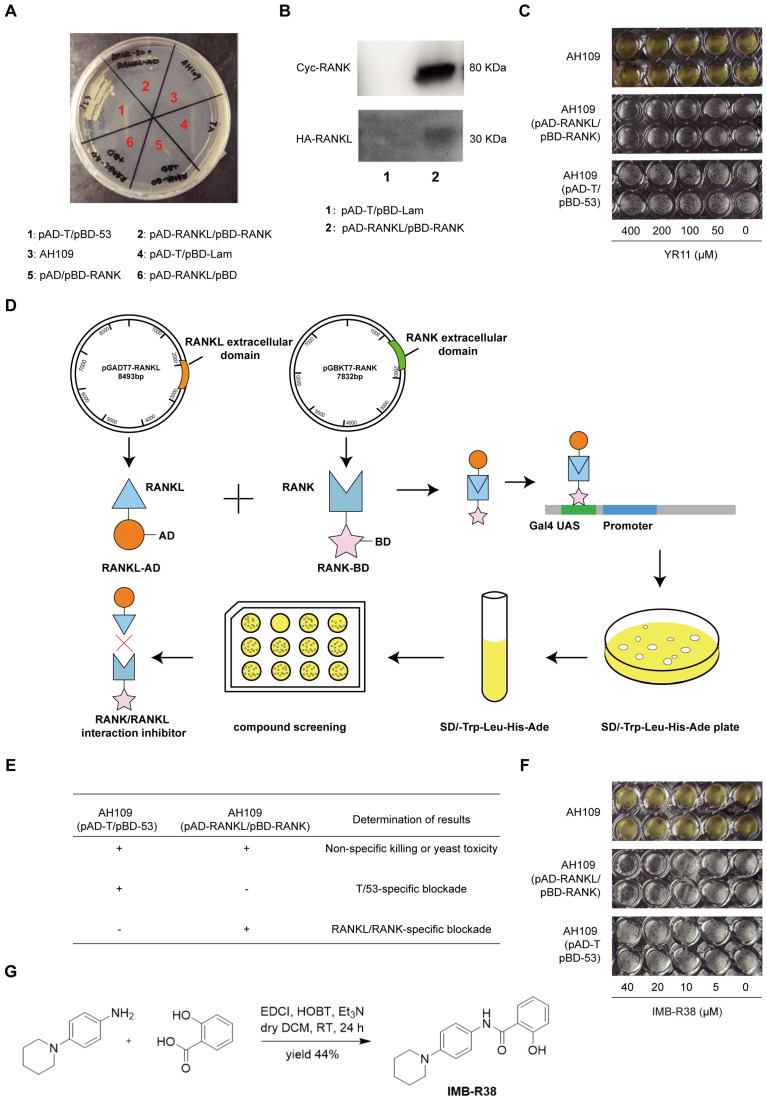
IMB-R38 blocks RANKL/RANK interaction in the yeast two-hybrid system. (**A**) Growth phenotypes of AH109 yeast strains transformed with different plasmids on SD/-Trp/-Leu/-His/-Ade dropout plates. The plasmid combinations for each group are as follows. 1: Positive control: AH109 (pAD-T and pBD-53). 2: AH109 yeast strains containing pAD-RANKL and pBD-RANK plasmids. 3: Blank control: untransformed AH109. 4: Negative control: AH109 (pAD-T and pBD-Lam). 5: To exclude self-activation: AH109 (pAD and pBD-RANK). 6: To exclude self-activation: AH109 (pAD-RANKL and pBD). (**B**) Western blot analysis confirmed the expression of Cyc-tagged RANK and HA-tagged RANKL fusion proteins in the AH109 (pAD-RANKL/pBD-RANK) stain. (**C**) Dose-dependent inhibition of AH109 (pAD-RANKL/pBD-RANK) growth by YR-11 (0, 50, 100, 200, 400 μM). (**D**) Schematic diagram illustrating the construction of the yeast two-hybrid system and the screening process for RANK/RANKL interaction inhibitors. (**E**) The screening criteria for identifying RANKL/RANK interaction inhibitors. (**F**) Specific inhibition of AH109 (pAD-RANKL/pBD-RANK) growth by IMB-R38 at concentrations of 0, 5, 10, 20, and 40 μM. (**G**) Synthetic route of IMB-R38.

To identify RANKL/RANK interaction inhibitors, we first established a RANKL/RANK interaction yeast two-hybrid system screening model. Two pairs of plasmids (pAD-RANKL/pBD-RANK or pAD-RANK/pBD-RANKL) were constructed and transformed into yeast AH109 cells. Growth of the transformed yeast cells on quadruple-dropout (SD/-Trp/-Leu/-His/-Ade) plates indicates activation of the reporter genes (ADE2, HIS3, LacZ) via RANKL-RANK binding. As shown in Figure 1A, only AH109 (pAD-RANKL/pBD-RANK) and positive control AH109 (pAD-T/pBD-53) successfully grew on quadruple-dropout plates, confirming the specific RANKL/RANK interaction. In contrast, no growth was observed for AH109 (pAD-RANK/pBD-RANKL), the negative control AH109 (pAD-T/pBD-Lam), or self-activation controls AH109 (pAD-RANKL/pBD or pAD/pBD-RANK) (Figure 1A), verifying the system’s specificity. Western blot analysis confirmed the expression of Cyc-tagged RANK and HA-tagged RANKL in AH109 (pAD-RANKL/pBD-RANK) (Figure 1B).

YR-11, a known OPG mimetic peptide that competitively inhibits RANKL/RANK binding, was used to validate the system. The results showed that YR-11 (0–400 μM) dose-dependently inhibited the growth of AH109 (pAD-RANKL/pBD-RANK) without affecting control strains (Figure 1C), confirming the specificity of the system and ruling out non-specific toxicity. The Z’ factor yielded a value of 0.77 (Figure 1C), well above the 0.50 threshold generally considered indicative of an excellent high-throughput screening (HTS) assay. These results collectively validate our yeast two-hybrid system as a reliable platform for studying RANKL-RANK interactions and screening inhibitors.

### 2.2. IMB-R38 Blocks RANKL/RANK Interaction in the Yeast Two-Hybrid System

Using the validated pAD-RANKL/pBD-RANK yeast two-hybrid system, we screened for RANKL/RANK interaction inhibitors (Figure 1D). Based on the screening criteria shown in Figure 1E, IMB-R38 was identified as a positive hit, which dose-dependently inhibited the growth of AH109 (pAD-RANKL/pBD-RANK) while showing no effects on either the parental AH109 strain or positive control AH109 (pAD-T/pBD-53) (Figure 1F). This pattern confirms that IMB-R38 disrupts the RANKL/RANK interaction rather than causing general toxicity or affecting the reporter system itself. The chemical structure and synthesis route of IMB-R38 are presented in Figure 1G, and its structure was confirmed by nuclear magnetic resonance (NMR) and mass spectrometry (MS) (Supplementary Figure S1).

### 2.3. IMB-R38 Inhibits RANKL-Induced Osteoclast (OC) Differentiation and Bone Resorption

**Figure 2:**
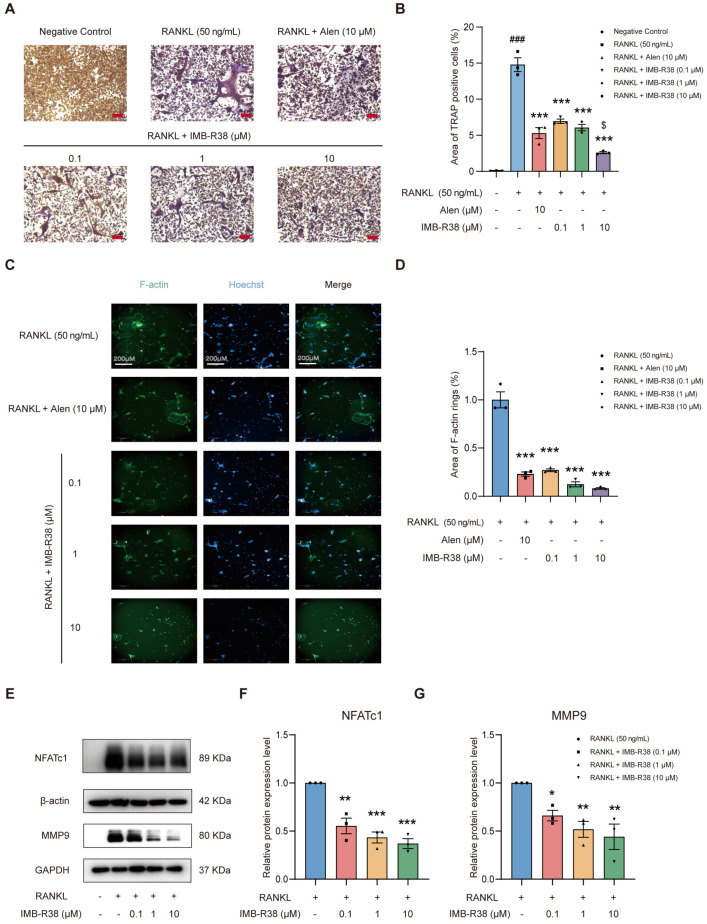
IMB-R38 suppresses RANKL-induced OC differentiation in vitro. (**A**–**D**) RAW264.7 cells were inoculated in 96-well plates, treated with 50 ng/mL of RANKL and IMB-R38 (0, 0.1, 1, and 10 μM) for 5 days. (**A**) Representative image of TRAP staining. Scale bar: 10 μm. (**B**) Quantification of the area of TRAP-positive cells. TRAP-positive multinucleated (nuclei ≥ 3) cells were counted as OCs. *n* = 3. (**C**) Representative images of RANKL-induced F-actin ring formation. Scale bar: 200 µm (**D**) Quantification of the area of F-actin rings. (**E**–**G**) RAW264.7 cells were seeded in 6-well plates and incubated with 50 ng/mL of RANKL and IMB-R38 (0, 0.1, 1, and 10 μM) for 24 h. Western blot analysis was performed to detect protein levels of NFATc1 and MMP9 in RAW264.7 cells. *n* = 3. All data are shown as the mean ± SEM. Significant differences were determined by One-way ANOVA. ### *p* < 0.001, RANKL group vs. DMSO control (no RANKL); * *p* < 0.05, ** *p* < 0.01, and *** *p* < 0.001, IMB-R38 group vs. RANKL group. $ *p* < 0.05, IMB-R38 group vs. the Alen group.

RANKL/RANK interaction is essential for OC differentiation and activity [15]. Thus, we evaluated the impact of IMB-R38 on OC differentiation and bone resorption. Tartrate-Resistant Acid Phosphatase (TRAP) (a validated marker for OCs [21]) staining showed that RANKL stimulation alone significantly promoted the differentiation of RAW264.7 into TRAP-positive multinucleated cells compared to the negative control (Figure 2A). In contrast to the RANKL-only group, IMB-R38 treatment dose-dependently reduced both the number and size of OCs (Figure 2A,B), directly inhibiting RANKL-mediated OC formation. F-actin rings are indispensable for OC motility and bone resorptive [22]. Phalloidin staining revealed robust F-actin ring formation in RANKL-stimulated OCs, whereas IMB-R38 dose-dependently disrupted F-actin ring assembly (Figure 2C,D), confirming its inhibitory effect on bone resorption. Notably, 10 μM IMB-R38 sharply suppressed RANKL-induced OC differentiation, which is even greater than that of 10 μM Alen.

As a master regulator, nuclear factor of activated T cells 1 (NFATc1) orchestrates OC differentiation and function by governing a transcriptional network [23]. Matrix metallopeptidase 9 (MMP9), a zinc-dependent type IV collagenase specifically expressed in OCs, modulates bone resorption by activating matrix-degrading pathways [24]. Western blot showed that IMB-R38 dose-dependently downregulated NFATc1 and MMP9 protein expression (Figure 2E–G). Taken together, these data suggested that IMB-R38 directly attenuates RANKL-induced OC differentiation and function in vitro.

### 2.4. IMB-R38 Promotes Osteoblast (OB) Differentiation In Vitro

**Figure 3:**
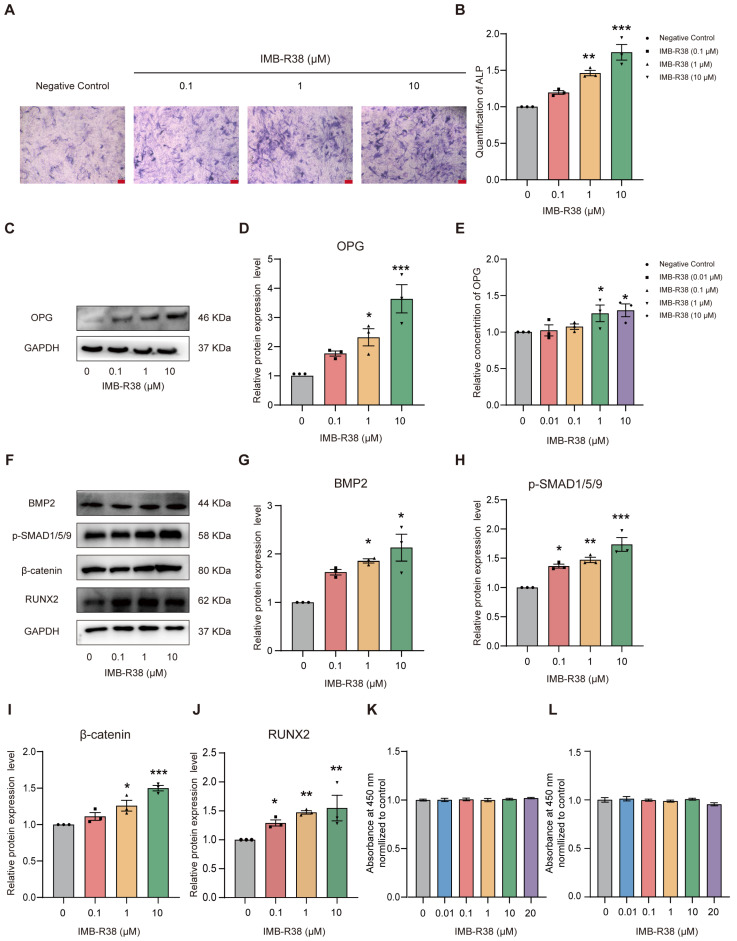
IMB-R38 promotes osteogenic differentiation by upregulating osteogenic protein expression in vitro. (**A**,**B**) C3H10^T1/2^ cells were cultured in osteogenic differentiation medium and treated with IMB-R38 (0, 0.1, 1, and 10 μM) for 7 days. (**A**) Representative images of ALP staining. Scale bar: 10 μm. (**B**) Quantification of ALP activity. *n* = 3. (**C**,**D**) MC3T3-E1 cells were treated with IMB-R38 at 0.1 μM, 1 μM, and 10 μM for 24 h. Protein levels of OPG were evaluated by Western blot and normalized to GAPDH. Representative images are shown. *n* = 3. (**E**) OPG secretion from MC3T3-E1 cells after treatment with IMB-R38 (0.01 μM, 0.1 μM, 1 μM, and 10 μM) for 48 h was measured by ELISA. *n* = 3. (**F**–**J**) Relative protein levels of bone morphogenetic protein 2 (BMP2), phospho-SMAD1/5/9 (p-SMAD1/5/9), β-catenin, and Runt-related transcription factor 2 (RUNX2) following 24 h treatment with IMB-R38 (0.1 μM, 1 μM, and 10 μM) were evaluated by Western blot and normalized to GAPDH. Representative images are shown. *n* = 3. (**K**,**L**) Cell viability assay. MC3T3-E1 and C3H10^T1/2^ cells were treated with increasing concentrations of IMB-R38 (0, 0.01, 0.1, 1, and 10 μM) for 48 h, respectively. All data are presented as mean ± SEM. Significant differences were determined by One-way ANOVA. * *p* < 0.05, ** *p* < 0.01, and *** *p* < 0.001 vs. control (without IMB-R38 treatment).

We next investigated the osteogenic potential of IMB-R38. Alkaline phosphatase (ALP) staining of the murine mesenchymal stem cell line (C3H10^T1/2^) cells under osteogenic differentiation conditions showed that IMB-R38 treatment resulted in a significant expansion of ALP-positive mineralization foci compared with the control group following 7 days of induction (Figure 3A,B). Given that OPG functions as a decoy receptor for RANKL to regulate bone remodeling [16], we assessed OPG expression levels. Western blot demonstrated that IMB-R38 upregulated OPG protein expression in a dose-dependent manner in C3H10^T1/2^ cells (Figure 3C,D), while quantitative enzyme-linked immunosorbent assay (ELISA) indicated that 1 μM and 10 μM IMB-R38 significantly promoted OPG secretion in mouse calvaria-derived preosteoblast cell line (MC3T3-E1) (Figure 3E).

BMP2 exhibits potent osteogenic activity by upregulating the expression of osteoblast differentiation markers [25]. Phospho-SMAD1/5/9 (p-SMAD1/5/9) are downstream proteins of the BMP2 signaling pathway related to OB differentiation [26]. RUNX2, a member of the Runx transcription factor family, is expressed in chondrocytes, osteoblast lineage cells, and thymocytes [27]. Osteoblast differentiation is promoted by canonical Wnt signaling via a mechanism whereby β-catenin/TCF1 directly activates the expression of Runx2 [28]. Together, these pivotal transcription factors play essential roles in both chondrocyte maturation and osteoblast differentiation [15,29,30]. Western blot showed that IMB-R38 upregulated the protein expression levels of canonical osteogenic markers, including BMP2 (Figure 3F,G), RUNX2 (Figure 3F,J), and proteins within their related signaling pathways, including p-SMAD1/5/9 (Figure 3F,H) and β-catenin (Figure 3F,I), in a dose-dependent manner. Furthermore, IMB-R38 exhibited no cytotoxicity within the concentration range of 0.01–20 μM (Figure 3K,L). Collectively, our data demonstrate that IMB-R38 potently promotes osteoblastic differentiation in both preosteoblastic and mesenchymal stem cell lineages in vitro, highlighting its dual regulatory role in osteogenesis and osteoclastogenesis inhibition.

### 2.5. The Anti-Osteoporosis Activity of IMB-R38 in the Dexamethasone (DXM)-Induced Osteogenic Zebrafish Model

**Figure 4:**
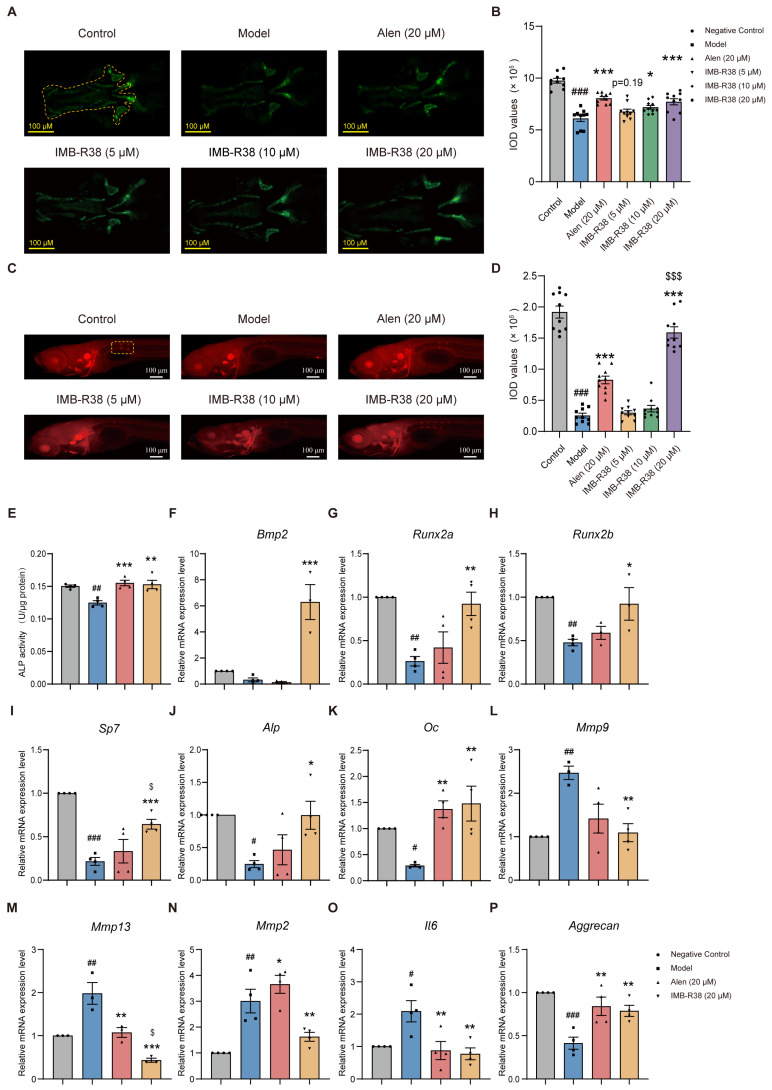
Effects of IMB-R38 on DXM-induced osteoporosis in two zebrafish models: AB strain and tg (OlaSp7:nlsGfp) cy25. Scale bar: 100 μm. (**A**) Fluorescence images of tg (OlaSp7:nlsGfp) cy25 zebrafish larvae under different treatments: control (no DXM). DXM alone (model), DXM + IMB-R38/Alen (positive control). The yellow dotted area is the analysis site. (**B**) Quantification of the integrated optical density (IOD) of skull fluorescence in tg (OlaSp7:nlsGfp) cy25 zebrafish larvae. (**C**) Alizarin red S (ARS)-stained images of AB strain wild-type zebrafish larvae treated with or without DXM, IMB-R38, or Alen. The yellow box indicates the region of interest (ROI) for analysis. Scale bar: 100 μm. (**D**) Quantification of the IOD of spine mineralization in ARS-stained zebrafish. (**E**) Quantification of ALP activity. (**F**–**P**) Relative mRNA expression levels including *Bmp2* (**F**), RUNX family transcription factor 2a (*Runx2a*) (**G**), RUNX family transcription factor 2b (*Runx2b*) (**H**), Sp7 transcription factor (*Sp7)* (**I**), *Alp* (**J**), Osteocalcin (*Oc*) (**K**), *Mmp9* (**L**), Matrix metallopeptidase 13 (*Mmp13*) (**M**), Matrix metallopeptidase 2 (*Mmp2*) (**N**), Interleukin-6 (*Il6*) (**O**), and *Aggrecan* (**P**) were detected by real-time quantitative PCR (RT-qPCR). Data are presented as mean ± SEM. Significant differences were determined by One-way ANOVA. # *p* < 0.05, ## *p* < 0.01, ### *p* < 0.001 vs. the control group (no DXM); * *p* < 0.05, ** *p* < 0.01, *** *p* < 0.001 vs. the model group (DXM only); $ *p* < 0.05 vs. the Alen group. $$$ *p* < 0.001 IMB-R38 group vs. the Alen group.

The anti-osteoporosis effect of IMB-R38 was evaluated in two dexamethasone (DXM)-induced zebrafish models. First, DXM-induced cy25 strain zebrafish was used, where green fluorescence reflects bone mass in the spine and vertebrae. DXM significantly reduced fluorescence intensity within the model group, confirming successful osteoporosis induction (Figure 4A,B). Subsequent treatment with IMB-R38 (5, 10, and 20 μM) resulted in an elevation in fluorescence intensity in a dose-dependent manner. Notably, the 10 μM and 20 μM groups exhibited substantial enhancement in bone mass restoration compared to the model group (Figure 4A,B). Importantly, IMB-R38 displayed superior pharmacological efficacy than the positive control, Alen, at an equivalent dosage of 20 μM (Figure 4A,B).

Second, in AB strain wild-type zebrafish, alizarin red staining with the red fluorescence intensity of the skull serving as an indicator of bone mas showed that DXM significantly reduced skull bone mass (Figure 4C,D). However, IMB-R38 dose-dependently increased red fluorescence intensity, indicative of enhanced skull bone mass (Figure 4C,D). Again, IMB-R38 outperformed Alen at equivalent doses (Figure 4C,D). Consistent with these findings, DXM reduced ALP activity, while IMB-R38 restored ALP activity levels comparable to Alen (Figure 4E).

Furthermore, to explore the mechanism underlying IMB-R38′s anti-osteoporotic effect in DXM-induced osteogenic zebrafish, RT-qPCR was conducted to analyze mRNA levels of osteoporosis-related genes. As shown in Figure 4F–K, DXM significantly downregulated the OB-specific genes (*Bmp2*, *Runx2a*, *Runx2b*, *Sp7*, *Alp*, and *Oc*) and markedly upregulated OC-specific genes (*Mmp2*, *Mmp9,* and *Mmp13*) compared with the control group. IMB-R38 notably reversed these DXM-induced changes, and it significantly upregulated OB-specific genes (*Bmp2*, *Runx2a*, *Runx2b*, *Sp7*, *Alp*, and *Oc*) and downregulated *Mmp2*, *Mmp9*, *Mmp13*, *and Il-6* compared with the model group (Figure 4F–O). In addition, IMB-R38 treatment significantly increased the *Aggrecan* mRNA level compared with the model group (Figure 4P). Collectively, these data indicate that IMB-R38 alleviates DXM-induced osteoporosis in DXM-induced zebrafish.

### 2.6. IMB-R38 Binds to RANK to Block the Interaction Between RANKL and RANK

**Figure 5:**
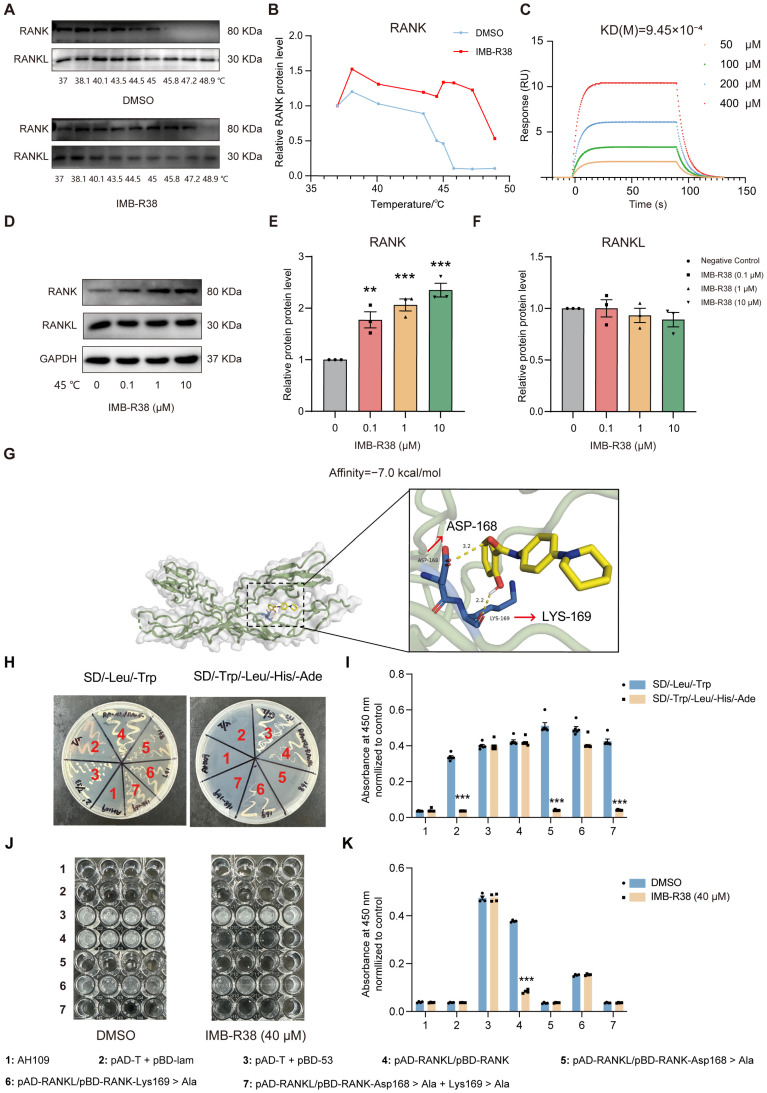
The mechanism by which IMB-R38 blocks RANKL-RANK interaction. (**A**) IMB-R38 improved the thermal stability of the RANK protein in RAW264.7 cells tested by cellular thermal shift assay (CESTA). (**B**) Quantification of CESTA band intensities by ImageJ-win64. (**C**) Surface plasmon resonance (SPR) analysis for the binding affinity of IMB-R38 interaction with RANK. The dissociation constant (KD) value was shown. (**D**–**F**) Dose-dependent effects of IMB-R38 administration (0.1, 1, 10 μM) toward RANKL/RANK expression levels at 45 °C in RAW264.7 cells. RANK protein level increased with RANKL levels unaffected. Protein band intensities were quantified by ImageJ-win64. *n* = 3. (**G**) Identification of critical amino acid residues in RANK mediating IMB-R38 interaction. (**H**,**I**) Growth phenotypes (**H**) and quantification (**I**) of AH109 yeast strains transformed with different plasmids on SD/-Leu/-Trp and SD/-Trp/-Leu/-His/-Ade dropout medium. (**J**,**K**) Growth phenotypes (**J**) and quantification (**K**) of IMB-R38 (40 μM)-mediated inhibition on AH109 growth transfected with different plasmids. Values are presented as means  ±  SEM. Significant differences were determined by One-way ANOVA (**E**,**F**), ** *p* < 0.01, and *** *p* < 0.001 vs. control (without IMB-R38 treatment) and Student *t* test analysis,*** *p* < 0.001 vs. SD/-Leu/-Trp (**I**) or treated without IMB-R38 (**K**).

We then investigated the mechanism by which IMB-R38 blocks the RANKL/RANK interaction using CETSA, which detects ligand-induced protein thermal stabilization [31]. As shown in Figure 5A,B, RANK protein levels in DMSO-treated groups decreased with increasing temperature (40.1–48.9 °C), while 10 μM IMB-R38 administration significantly slowed RANK degradation at the same temperatures. At a fixed temperature of 45 °C, IMB-R38 increased RANK protein stability in a dose-dependent manner (0–10 μM) (Figure 5D–F). However, IMB-R38 failed to enhance RANKL protein stability (Figure 5D–F). These data suggested that IMB-R38 specifically binds with RANK rather than RANKL. SPR is a preeminent method for label-free investigations of target–ligand interactions [32]. SPR results confirmed that IMB-R38 bound to RANK in a concentration-dependent fashion (50–400 μM) with a dissociation constant (KD) of 9.45 × 10^−4^ M (Figure 5C) but did not bind to RANKL (Supplementary Figure S2C).

Molecular docking results predicted that IMB-R38 forms intermolecular hydrogen bonds with Asp168 and Lys169 of RANK, resulting in moderate binding affinity (Figure 5G). To verify whether Asp168 and Lys169 of RANK are key amino acids for RANK/RANKL interaction, three pBD-RANK site-directed mutant plasmids were generated (pBD-RANK-Asp168 > Ala, pBD-RANK-Lys169 > Ala, and pBD-RANK-Asp168 + Lys169 > Ala). Each mutant plasmid was co-transformed with pAD-RANKL plasmid into yeast AH109 cells, respectively. Interestingly, Asp168 mutant alone and Asp168 + Lys169 double mutant lead to no strain growth on the SD/-Trp/-Leu/-His/-Ade quadruple-dropout medium (Figure 5H,I). In contrast, the Lys169 single mutant did not affect strain growth (Figure 5H,I). These results demonstrated that Asp168, but not Lys169, is a key amino acid for RANK/RANKL interaction. Furthermore, IMB-R38 exerted an inhibitory effect only on AH109 (pBD-RANK + pAD-RANKL) but not on strains harboring other plasmids (Figure 5J,K). Overall, these data demonstrate that IMB-R38 specifically binds with RANK to block the RANKL/RANK interaction.

### 2.7. IMB-R38 Inhibits RANKL-Stimulated Activation of MAPK Signaling Pathways

**Figure 6:**
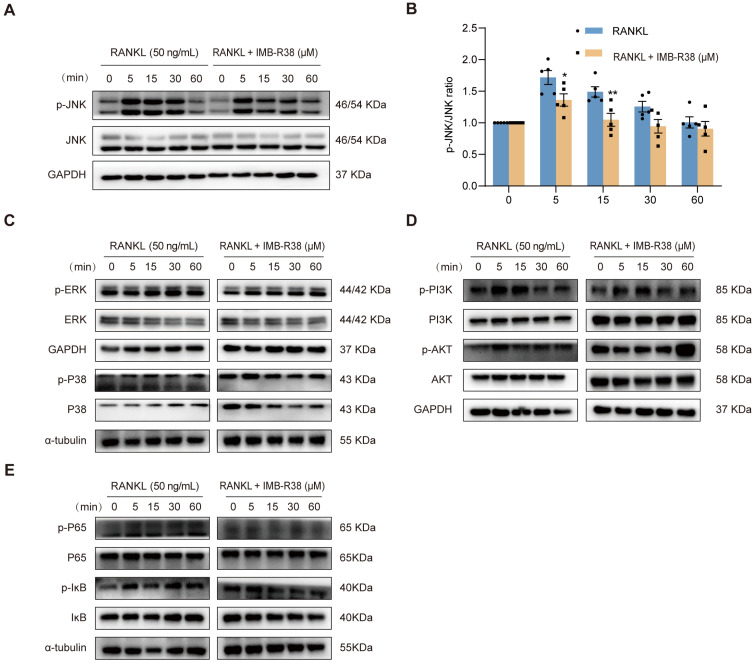
IMB-R38 disrupts RANKL-induced OCs differentiation by inhibiting MAPK signaling pathways. (**A**–**E**) RAW264.7 cells were treated with or without 10 μM IMB-R38 for 6 h and stimulated with 50 ng/mL of RANKL for 0, 5, 15, 30, and 60 min. (**A**,**B**) Total proteins were extracted for Western blot and normalized to GAPDH, and the relative ratio of p-JNK/JNK was calculated by ImageJ-win64 software. *n* = 3. (**C**–**E**) Western blot was performed to detect the expression levels of p-ERK/ERK, p-P38/P38, p-PI3K/PI3K, P-AKT/AKT, p-P65/P65, and p-IĸB/IĸB. Protein expression was normalized to GAPDH or α-tubulin. Data are expressed as the mean ± SEM; significant differences were determined by Student *t* test analysis. * *p* < 0.05 and ** *p* < 0.01 in contrast with the RANKL group.

The RANKL/RANK interaction initiates multiple downstream signaling cascades, with the MAPK pathway serving as an indispensable mediator in RANKL/RANK-driven OC proliferation, differentiation, and bone resorption [33]. To explore the regulatory impact of IMB-R38 on RANKL-triggered MAPK signaling, a Western blot assay was conducted. IMB-R38 significantly attenuated JNK phosphorylation, a key event in MAPK pathway activation, without altering total JNK protein levels (Figure 6A,B). In contrast, as shown in Figure 6C, IMB-R38 exerted no significant effect on the phosphorylation or total expression of other MAPK family members (P38 and ERK) or on the AKT and NF-κB signaling pathways (Figure 6D,E). Collectively, these results indicate that IMB-R38 inhibits osteoclastogenesis by selectively disrupting the RANKL/RANK-induced MAPK signaling axis.

## 3. Discussion

Osteoporosis is a severe chronic disease caused by a bone remodeling imbalance [1]. The binding of RANKL to RANK promotes OC differentiation and bone resorption [15]. When OC-mediated bone resorption exceeds OB-mediated bone formation, bone remodeling imbalance and osteoporosis occur [13]. It is a consensus that inhibiting RANKL and RANK interaction is an effective strategy for anti-osteoporosis drug development [34]. Denosumab, an anti-RANKL monoclonal antibody, has been approved for the treatment of postmenopausal osteoporosis, giant cell tumor of bone [35,36,37], and bone cancer metastasis [38]. However, due to its potential immune-related side effects and high cost [39,40], there remains a need for safer and cheaper drugs. Some small-molecule compounds targeting RANKL/RANK interaction were reported in recent years. For example, AS2676293 was identified as a small-molecule RANKL inhibitor that treats bone metastasis by inhibiting both osteoclastic bone resorption and tumor migration to bone [41]. Xu et al. revealed that both ellagic acid and niloticin block RANKL/RANK interaction and suppress RANKL-induced osteoclastogenesis [42,43]. However, these compounds above only suppress OC differentiation, and their effects on bone formation have not been thoroughly evaluated. Overall, these data above demonstrate that inhibiting RANKL/RANK interaction is an effective and potent strategy for anti-osteoporosis drug development.

In this study, IMB-R38 was identified as a novel RANK/RANKL interaction inhibitor using a yeast two-hybrid system. IMB-R38 displayed a novel structure distinct from existing RANKL/RANK inhibitors. RANKL and RANK interaction mainly promotes OC differentiation and bone resorption, resulting in osteoporosis. We demonstrated that IMB-R38 could directly inhibit OC differentiation and bone resorption in RANKL-induced RAW264.7 cells, which might underlie its anti-osteoporotic activity. The mechanistic study revealed that IMB-R38 exhibits moderate affinity for RANK (KD = 9.45 × 10^−4^ M) but not RANKL, distinguishing it from typical RANKL/RANK inhibitors such as denosumab and AS2676293. Molecular docking simulation predicted that Asp168 and Lsy169 are critical amino acids for IMB-R38-RANK binding, while further experiments confirmed that Asp168 but not Lys169 is essential for RANKL/RANK interaction. Our results also demonstrate that IMB-R38 occupies key amino acid Asp168, thereby blocking RANKL/RANK interaction and confirming its role as a RANKL/RANK inhibitor.

The MAPK pathway is activated upon RANKL/RANK binding. IMB-R38 specifically inhibited JNK phosphorylation without affecting the PI3K/AKT, P65/IκB, or ERK/P38 pathways. As a member of the MAPK gene family, JNK plays a crucial role in inflammatory responses. Interestingly, IMB-R38 treatment significantly decreased mRNA levels of *Mmp9*, *Mmp13*, *Mmp2,* and *Il-6*, which play a vital role in both osteoporosis and inflammation [44,45,46]. Given that osteoporosis is recognized as an inflammatory disease, IMB-R38 may ameliorate osteoporosis by alleviating inflammation. JNK is indispensable for signal transduction and closely associates with neurodegenerative diseases, cancer, diabetes, and so on. As a JNK inhibitor, SP600125 has been reported to alleviate osteoarthritis in mice [47]. Aggrecan, a crucial structural protein within the extracellular matrix of cartilage, undergoes degradation in osteoarthritis [48,49]. Intriguingly, IMB-R38 treatment significantly increased the *Aggrecan* mRNA level compared with the model group, suggesting its therapeutic potential for osteoarthritis, although further investigation is required.

OBs-driven bone formation is equally essential to osteoporosis [50]. In this study, IMB-R38 treatment upregulated key OB-related genes, including OPG, RUNX2, β-catenin, pSMAD1/5/9, and BMP2, suggesting its potential to promote osteogenic differentiation. Importantly, IMB-R38 significantly enhanced bone formation in a dexamethasone (DXM)-induced osteoporotic zebrafish model. Collectively, these findings demonstrate that IMB-R38 could promote osteogenesis, thereby improving the balance of bone remodeling. However, the precise molecular mechanisms underlying IMB-R38-mediated bone metabolism, particularly how it promotes osteogenesis and its function in OB-OC crosstalk, remain to be elucidated.

Taken together, this study identifies IMB-R38 as a novel RANKL/RANK inhibitor that functions as a dual regulator of bone formation and bone resorption in vitro and zebrafish osteoporosis models in vivo, with superior efficiency compared to Alen. Further evaluation in mammalian models will also be necessary to fully evaluate the anti-osteoporotic potential of IMB-R38.

## 4. Materials and Methods

### 4.1. Synthesis of IMB-R38

2-hydroxy-*N*-(4-(piperidin-1-yl)phenyl)benzamide (named IMB-R38) was synthesized from 4-(piperidin-1-yl)aniline and 2-hydroxybenzoic acid, yielding a faint yellow oil. The detailed synthesis steps are as follows: A solution of EDCI (359 mg, 1.87 mmol) dissolved in dry DCM (10 mL) was mixed with HOBT (253 mg, 1.87 mmol) and Et_3_N (0.87 mL, 6.24 mmol) at room temperature (RT) and stirred until dissolved. At 0 °C, 2-hydroxybenzoic acid (215.5 mg, 1.56 mmol) and 4-(piperidin-1-yl)aniline (275 mg, 1.56 mmol) were added to the mixture, which was then stirred at RT for 24 h. The solvent was removed under reduced pressure. The crude product was purified on silica gel column chromatography (PE/EA = 10:1 as eluent) to obtain IMB-R38 (205 mg, 44% yield) as a faint yellow oil. The chemical structure of IMB-R38 was confirmed by NMR and MS. ^1^H NMR (400 MHz, DMSO-*d*_6_) δ 12.12 (s, 1H), 10.22 (s, 1H), 7.99–7.97 (s, 1H), 7.53–7.49 (m, 2H), 7.45–7.41 (m, 1H), 6.97–6.91 (m, 4H), 3.12–3.09 (m, 4H), 1.65–1.59 (m, 4H), 1.55–1.49 (m, 2H). ^13^C NMR (100 MHz, DMSO-*d*_6_) δ 167.08, 159.64, 149.20, 134.08, 129.70, 129.00, 122.83, 119.31, 117.77, 117.21, 116.38, 50.32, 25.72, 24.34. HRMS-ESI (m/z): Calcd. for C_18_H_20_N_2_O_2_[M + H]^+^: 296.1526, found: 297.1666.

### 4.2. Plasmid Construction

DNA fragments encoding RANKL (GenBank: AAB86812.1, amino acids 143-317, nucleotides 579-1103) and RANK (GenBank: AAB86810.1, amino acids 26-211, nucleotides 134-676) were inserted into the pGADT7 plasmid (activation domain, AD) with *Nde*I and *BamH*I, which resulted in in-frame fusions of RANKL or RANK with the GAL4 transcription activation domain, generating recombinant plasmids pAD-RANKL and pAD-RANK, respectively. Similarly, the same RANKL and RANK coding sequences were inserted sequentially into the pGBKT7 plasmid (GAL4 DNA-binding domain, BD) via *Nde*I/*BamH*I digestion, which resulted in in-frame fusions of RANKL or RANK with the GAL4 DNA-binding domain, yielding recombinant plasmids pBD-RANKL and pBD-RANK, respectively. All plasmids were synthesized by the Genscript Biotech Corporation (Nanjing, China). The control plasmids (pBD-53, pBD-lam, and pAD-T) were maintained by our laboratory.

### 4.3. Yeast Two-Hybrid Assay

Two plasmid combinations, pAD-RANK/pBD-RANKL and pAD-RANKL/pBD-RANK, were obtained. The pAD-RANKL plasmid was co-transformed with pBD-RANK into yeast AH109 cells to obtain AH109 (pAD-RANKL + pBD-RANK), while pAD-RANK and pBD-RANKL plasmids were co-transformed into yeast AH109 to generate AH109 (pAD-RANK + pBD-RANKL). Control strains were similarly generated, including AH109 (pAD-RANKL + pBD) and AH109 (pAD + pBD-RANK) to monitor self-activation, along with positive control AH109 (pAD-T + pBD-53) and negative control AH109 (pAD-T + pBD-lam). All transformants were initially selected on SD/-Leu/-Trp dropout plates (1811547A, Takara, Japan) at 30 °C for 3–4 days. Only the AH109 strain containing both pAD and pBD is able to grow on the double-dropout plates. Subsequently, these transformants were streaked onto SD/-Trp/-Leu/-His/-Ade quadruple-dropout plates (S6120, Solarbio, Beijing, China) at 30 °C for 3–4 days, where growth indicated a successful interaction between the fusion proteins through the activation of the reporter genes. This growth phenotype specifically resulted from the functional reconstitution of the GAL4 transcription factor driven by RANKL/RANK binding.

### 4.4. Assessment of the Yeast Two-Hybrid System

Protein expressions in the yeast were confirmed by Western blot. To validate the suitability of this yeast two-hybrid system for high-throughput drug screening, YR-11 (YLEIEFSLKHR, synthesis by Sangon Biotech Co., Ltd., (Shanghai, China), a known OPG-like peptidomimetics that can competitively inhibit RANKL/RANK interaction was used as a positive control [51]. System robustness was quantitatively assessed by calculating the Z’ factor [52].

### 4.5. RANKL/RANK Interaction Inhibitor Screening

HTS was performed using the validated yeast two-hybrid protocol [53] with three critical yeast strains: the parental AH109 control, positive interaction control AH109 (pAD-T/pBD-53), and our built strain AH109 (pAD-RANKL/pBD-RANK). Yeast strains grown in SD/-Trp/-Leu/-His/-Ade quadruple-dropout medium were seeded into a 96-well plate, followed by compound addition and incubation at 30 °C for 3–4 days, and the growth inhibition was monitored. The following criteria were adopted for the HTS of RANKL/RANK interaction inhibitors (Figure 1E). In brief, compounds that inhibit the growth of AH109 are identified as antifungal agents. Compounds that inhibit both the growth of positive control AH109 (pAD-T/pBD-53) and AH109 (pAD-RANKL/pBD-RANK) in quadruple-dropout (SD/-Trp/-Leu/-His/-Ade) medium are identified as non-specific RANKL/RANK protein–protein interaction (PPI) disruption or GAL4 expression disruption. Meanwhile, compounds that only suppress the AH109 (pAD-RANKL/pBD-RANK) growth but have no effect on the positive control and AH109 yeast cells are considered positive RANKL/RANK interaction inhibitors.

### 4.6. Expression and Purification of RANKL and RANK Proteins

The expression and purification of RANKL and RANK proteins were performed with reference to the protocol described by Zhang et al. [54]. Briefly, the ectodomain of RANKL (amino acid 158–316) was cloned into the pGEX-4T-1 plasmid via *BamH*I and *Sma*I sites to generate the GST-RANKL fusion protein expression plasmid. The extracellular segment of RANK (amino acid 26–210) was inserted into the pET28a (+) plasmid with *Nde*I and *BamH*I sites to generate the His-RANK fusion protein expression plasmid. All plasmids were synthesized by the Genscript Biotech Corporation (Nanjing, China). Then the two plasmids were transformed into *E. coli* competent cells BL21 (DE3) for RANKL and RANK protein expression and purification. Detailed purification protocols are provided in the Supplementary Methods.

### 4.7. Extraction of Total Yeast Proteins

AH109 (pAD-RANKL and pBD-RANK) and AH109 (pAD-T and p-BD-Lam) strains were inoculated into 4 mL of SD/-Trp/-Leu/-His/-Ade dropout medium at 30 °C with shaking at 220 rpm for 48 h. Cells were harvested by centrifugation at 8000× *g* for 2 min at 4 °C, then resuspended in 1 mL of sterile water. After centrifugation, the pellet was resuspended in yeast protein extraction reagent (9780, Takara, Osaka, Japan) and incubated in a warm water bath for 30 min. The supernatants were mixed with 2× protein loading buffer (P1040, Solarbio, Beijing, China) and heated in a metal bath (MK-20, Allsheng, Hangzhou, China) at 100 °C for 10 min for subsequent Western blot analysis.

### 4.8. Cell Culture

C3H10^T1/2^ and MC3T3-E1 cells were procured from Zebra Biotechnology Co. Ltd. (Changsha, China). C3H10^T1/2^ cells were cultured in Earle’s balanced salt solution-containing MEM (MEM, Hyclone) supplemented with 10% fetal bovine serum (FBS, Gibco, Carlsbad, CA, USA) and 100 U/mL of P/S (PB180120, Pricella, Wuhan, China). MC3T3-E1 cells were cultured in α-minimum essential medium (α-MEM, Hyclone, Waltham, MA, USA) supplemented with 10% FBS and 100 U/mL P/S. The osteogenic induction medium 1 for MC3T3-E1 cells was prepared with α-MEM containing 10% FBS, 100 U/mL of P/S, 50 mg/mL of L-Ascorbic Acid (A0537, TGI, Shanghai, China), and 10 mM β-glycerophosphate (G9422-50G, Sigma-Aldrich, St. Louis, MO, USA). For C3H10^T1/2^ osteogenesis, 10 nM dexamethasone was additionally added in osteogenic induction medium 1 (osteogenic induction medium 2). 

The murine macrophages RAW264.7 were obtained from the Cell Resource Center for Basic Medicine, Chinese Academy of Medical Sciences (CAMS, Beijing, China), and cultured in Dulbecco’s modified Eagle medium (DMEM, Hyclone) supplemented with 10% FBS and 100 U/mL of P/S. RAW264.7 cells simulated differentiation into OCs using α-MEM containing 50 ng/mL of RANKL (0121233, PeproTech, Cranbury, NJ, USA) with 10% FBS and 100 U/mL of P/S (osteoclastic induction medium). All cell lines were maintained in a humidified 37 °C incubator with 5% CO_2_.

### 4.9. Cell Viability

Cytotoxicity was evaluated using the cell counting kit-8 (CCK8) according to the manufacturer’s protocol (CA1210, Solarbio, Beijing, China). MC3T3-E1 cells were seeded into 96-well plates at a density of 1 × 10^4^ cells/well and pre-incubated for 24 h to allow cell attachment. Subsequently, cells were treated with serial concentrations of IMB-R38 (0, 0.01, 0.1, 1, and 10 μM) for 48 h. Absorbance at 450 nm was then measured using a microplate reader (EnVision 2104, PerkinElmer, Shelton, CT, USA).

### 4.10. ALP Staining Assay

ALP is a well-established phenotypic marker for OB activity and bone formation processes [55,56]. ALP staining was performed as in a previous study [57]. C3H10^T1/2^ cells were seeded in 96-well plates and cultured in osteogenic induction medium 2 in the presence or absence of IMB-R38 (0.1, 1, or 10 μM) for 7 days. Cells were washed three times with PBS, fixed with 4% paraformaldehyde (PFA, BL539A, Biosharp, Heifei, China) for 30 min at RT, and incubated with the BCIP/NBT ALP development kit (Beyotime Biotechnology, Shanghai, China) in the dark for 1 h. Stained cells were imaged using an inverted phase-contrast microscope (DMIL, Leica, Wetzlar, Germany).

### 4.11. TRAP Staining and Quantification

TRAP is a specific histochemical marker for OC identification [58,59]. RAW264.7 cells were seeded in 96-well plates at a density of 5 × 10^3^ cells/well. After attachment, cells were cultured in osteoclastic induction medium with or without IMB-R38 (0.1, 1, and 10 μM) for 5 days. Alen (Merck & Co, Rahway, NJ, USA) was used as a positive control. Fresh medium was replaced every 48–72 h during the culture period. Then the cells were fixed with 4% PFA for 30 min at RT, followed by TRAP staining using a leukocyte acid phosphatase kit (387A, Sigma-Aldrich). Stained cells were imaged using an inverted phase-contrast microscope (DMIL, Leica, Wetzlar, Germany). Multinucleated TRAP-positive cells (≥3 nuclei) were counted as mature.

### 4.12. F-Actin Ring Staining

F-actin ring formation is essential for osteoclast-mediated bone resorption and serves as a critical morphological indicator of mature OCs [60]. RAW264.7 cells were treated as described in the TRAP staining assay. Cells were permeabilized with PBS containing 0.5% Triton X-100 for 10 min, washed three times with PBS, and incubated with TRITC-conjugated phalloidin (working concentration, 80–100 nM; Solarbio, Beijing, China) for 1 h in the dark. Nucleus was labelled with Hoechst 33342 (B8040, Solarbio, Beijing, China) for 5 min. Fluorescent images were acquired using a high-content imaging system (Opetta CCS, PerkinElmer, Shelton, CT, USA).

### 4.13. Western Blot Assay

For signaling pathway research, RAW264.7 cells in 6-well plates were pretreated with IMB-R38 (10 μM) for 6 h prior to stimulation with 50 ng/mL of RANKL for defined intervals (0, 5, 15, 30, and 60 min). To analyze the expression of OC marker genes, RAW264.7 cells were treated with IMB-R38 (0, 0.1, 1, and 10 μM) in osteoclastic induction medium for 5 days.

For the assessment of osteogenesis-related gene expression, MC3T3-E1 or C3H10^T1/2^ cells were seeded in 6-well plates and cultured in osteogenic induction medium 1 or 2 with IMB-R38 (0, 0.1, 1, or 10 μM) for 3 days.

After the treatment above, cells were lysed in RIPA buffer (Beyotime Biotechnology, Shanghai, China) supplemented with 1 mM PMSF (ST506, Beyotime Biotechnology, Shanghai, China) for 30 min on ice. Lysates were centrifugated and protein concentrations of the supernatants were determined using the Pierce™ BCA Protein Assay Kit (Thermo, Waltham, MA, USA). Proteins were separated by 10% SDS-PAGE and transferred to PVDF membranes (ISEQ00010, Millipore, Bedford, MA, USA). Membranes were blockaded with 5% non-fat milk for 1 h at RT, incubated with primary antibodies and HRP-conjugated secondary antibodies, and visualized using the Tanon 5200 Chemiluminescent Imaging System (Tanon model 5200, Shanghai, China). Detailed antibody information is provided in Supplementary Table S1. All uncropped membranes were shown in Supplementary Figure S3.

### 4.14. OPG Secretion Assay

MC3T3-E1 cells were seeded in 12-well plates and treated with IMB-R38 (0, 0.01, 0.1, 1, or 10 μM) for 48 h. OPG secretion was quantified using a commercial ELISA kit (Nanjing Sen Bei Jia Biotechnology Co., Ltd., Nanjing, China). Absorbance at 450 nm was determined using a microplate reader and OPG concentrations were calculated by interpolation from a standard curve.

### 4.15. Evaluation of Anti-Osteoporosis Efficacy in Wild-Type AB Strain Zebrafish

The animal study was approved by the Institutional Animal Care and Use Committee of Hunter Biotechnology, Inc. (approval number IACUC-2024-9937-01).

The DXM-induced osteoporotic wild-type AB strain zebrafish model was established as previously described [61]. Briefly, zebrafish at three days post-fertilization (3 dpf) were cultured at 28 °C in fish culture water, which consisted of 200 mg/L of instant sea salt and 50–100 mg/L of CaCO_3_, with a conductivity range of 450–550 μS/cm and a pH range of 6.5–8.5. Wild-type AB strain zebrafish at 3 days post-fertilization (3 dpf) were randomly allocated into six-well plates (30 zebrafish/well). For the normal control group, the zebrafish were treated with DMSO. The model group was exposed to DXM (1.5 μM, Lot No. C2110208, Aladdin Biochemical Technology Co., Ltd., Shanghai, China) plus DMSO. As for the experimental group, the zebrafish were treated with DXM (1.5 μM) and IMB-R38 at different concentrations (5, 10, and 20 μM) for 96 h. And the positive control group was exposed to DXM (1.5 μM) and Alen (20 μM). No mortalities were observed in any of the groups after 3 days of exposure. At the end of the experiment, the zebrafish were either subjected to staining or harvested for a RT-qPCR assay.

AB strain zebrafish were euthanized and fixed in a 4% paraformaldehyde solution for a duration of 2 h. Subsequently, the fixed larvae were stained with an alizarin red S (ARS, C12071107, Macklin Inc., Shanghai, China) solution. Ten ARS-stained zebrafish were randomly selected and imaged using a stereomicroscope (SZX7, Olympus, Tokyo, Japan). The integrated optical density (IOD) of ARS-stained spine mineralization was quantified using NIS-Elements D 3.20 advanced image processing software. For the ALP activity in zebrafish, the supernatant of total zebrafish lysates was analyzed using an ALP assay kit (Beyotime Biotechnology, Shanghai, China).

Total RNA from each group (30 tails per group) was extracted using TRIZOL reagent (Transgen Biotech, Beijing, China), and cDNA was synthesized via reverse transcription using the RevertAid First Strand cDNA Synthesis Kit (Thermo Scientific, Waltham, MA, USA) according to the manufacturer’s instructions. RT-qPCR was performed utilizing the QuantiNova SYBR Green PCR Kit (Qiagen, Hilden, Germany) to quantify the mRNA expression levels of genes including *BMP2*, *Runx2a*, *Runx2b*, *Sp7*, *Alp*, *Oc*, *Mmp9*, *Mmp13*, *Mmp2*, *Il6*, and *Aggrecan*. *β-actin* was used as the internal control. The sequences of the primers are provided in Supplementary Table S2.

### 4.16. Evaluation of the Anti-Osteoporosis Effect in the Transgenic Green Fluorescent Zebrafish

The DXM-induced osteoporotic model was established using transgenic green fluorescent zebrafish tg (OlaSp7:nlsGfp) cy25, as previously described [61]. Zebrafish at 3 dpf were maintained in 6-well plates (30 larvae/well, 3 mL medium/well) and treated for 96 h as follows: DXM (10 μM), DXM (10 μM) + IMB-R38 (5, 10, 20 μM), or DXM (10 μM) + Alen (20 μM). After treatment, 10 larvae per group were imaged using a fluorescence microscope (AZ100, Nikon, Tokyo, Japan), and the fluorescence IOD of the zebrafish cephalic bone region was quantified using NIS-Elements D 3.20 imaging processing software.

### 4.17. SPR

SPR measurements were conducted using an OpenSPR system (Wayen Biotechnologies, Inc., Shanghai, China) to assess the binding affinity and kinetic properties between IMB-R38 and RANKL/RANK. Specifically, RANKL and RANK proteins were dissolved in sodium acetate buffer and immobilized onto a dextran-coated sensor chip (Reichert, NY, USA) via amine coupling. For affinity determination, IMB-R38 was prepared at serial concentrations (50, 100, 200, 400 μM) in PBST-D running buffer (PBST containing 0.5% DMSO, pH 7.4) and injected over the protein-immobilized chip surface using a Reichert 2 SPR system at a flow rate of 30 μL/min. Each injection included a 30 s association phase followed by a 150 s dissociation phase. Sensorgram data were analyzed using TraceDrawer software 1.7.1 (Reichert, Depew, NY, USA) with reference cell subtraction and buffer blank adjustment, and kinetic parameters were derived by global fitting to a 1:1 Langmuir binding model.

### 4.18. CESTA

The CETSA is a biophysical method for evaluating the binding efficacy of small-molecule ligands to their target proteins by measuring the thermal stabilization of ligand-induced target proteins under native cellular conditions [62]. RAW264.7 cells were treated with IMB-R38 or DMSO for 6 h, and cell pellets were subjected to thermal challenge in a PCR thermocycler. The relative abundance of RANK and RANKL was determined by Western blot. Detailed methods are provided in the Supplementary Materials.

### 4.19. Molecular Docking

PyMOL (The PyMOL Molecular Graphics System, version 3.0.3) was utilized to preprocess the RANK (pdb_00003me4) [63] and IMB-R38 structure for molecular docking. Briefly, the protein structure of RANK was imported into AutoDockTools (ADT, version: 1.5.7), and then co-crystalized water and irrelevant molecules were removed, and polar hydrogens were added. The 3D structure of IMB-R38 was also imported into the AutoDock Tools-1.5.7 software. The active site of RANK was determined as 70 Å × 110 Å × 70 Å. Molecular docking was performed using Auto Dock Vina with 10 independent runs, and a schematic diagram of the RANK-IMB-R38 interaction was generated.

### 4.20. Site-Directed Mutation

The Asp168 and Lys169 within the pBD-RANK plasmid were mutated to Ala, respectively, generating the mutant plasmids designated as pBD-RANK-Asp168 > Ala, pBD-RANK-Lys169 > Ala, and pBD-RANK-Asp168 + Lys169 > Ala. The mutant plasmids were synthesized by Genscript Biotech Corporation (Nanjing, China). Each of the three plasmids was co-transformed with pAD-RANKL into yeast AH109 cells. The transformants were inoculated on SD/-Leu/-Trp dropout medium at 30 °C for 3–4 days, followed by further cultivation in SD/-Trp/-Leu/-His/-Ade quadruple-dropout medium at 30 °C for an additional 3–4 days, which was in accordance with previous descriptions in Section 4.3. This growth phenotype on quadruple-dropout medium indicated specific binding between RANKL and RANK.

### 4.21. Statistical Analysis

All quantitative data are presented as mean ± standard error of the mean (SEM). Statistical analyses were performed using GraphPad Prism 8.0 software (San Diego, CA, USA). A two-tailed Student *t* test was used to compare the significance between two groups. For comparisons among three or more groups, One-way analysis of variance (ANOVA) with Tukey’s post hoc test was applied. *p* < 0.05 was considered statistically significant.

## Data Availability

The data that support the findings of this study are available from the corresponding author, Yanni Xu, upon reasonable request.

## Supplementary Materials

The following supporting information can be downloaded at: https://www.mdpi.com/article/10.3390/ijms262412151/s1.
