# Supplementary material for: A Novel RANKL/RANK Inhibitor IMB-R38 Inhibits Osteoporosis Through Regulating Bone Metabolism
Source: Int J Mol Sci. 2025 Dec 17;26(24):12151. doi: 10.3390/ijms262412151 (PMC12733451; doi:10.3390/ijms262412151)
Supplement: Supplementary file 1 [file ijms-26-12151-s001.zip › ijms-3949905-supplementary.pdf]

A novel RANKL/RANK inhibitor IMB-R38 inhibits osteoporosis through regulating bone metabolism

Yuyan Zhang#, Xinwei Wei#, Ren Sheng, Guijun Yang, Xiaowan Han, Jingrui Wang, Chao Liu, Shunwang Li, Lijuan Lei, Weilian Jiang, Yang Lun, Shuyi Si, Jing Zhang*, Yanni Xu*

State Key Laboratory of Bioactive Substance and Function of Natural Medicines, NHC Key Laboratory of Microbial drugs, National Center for New Microbial Drug Screening, Institute of Medicinal Biotechnology, Chinese Academy of Medical Sciences & Peking Union Medical College (CAMS&PUMC), Tiantan Xili 1#, Beijing, China, 100050.

# These authors contributed equally to this word.

*Correspondence: jingjingz@imb.pumc.edu.cn (J.Z.); xuyanni2010@imb.pumc.edu.cn (Y.X.).

**Supplemental Materials and Methods:**

Supplementary Methods.

Supplementary Table S1-S2;

Supplementary Figure S1-S3.

## 1. Supplementary Methods

### *1.1 Expression and purification of RANKL and RANK protein*

The BL21 (DE3) harboring the pGEX-4T-RANKL plasmid was cultured in LB medium containing 100 μg/ml ampicillin at 37 °C with agitation (200 rpm) until optical density at 600 nm ($OD_{600}$) reached 0.6~0.8. Following mid-log phase growth, recombinant protein expression was induced by adding 0.05 mM isopropyl β-D-thiogalactopyranoside (IPTG) (I8070, Solarbio, Beijing, China) and incubating at 22 °C overnight. Cells were then collected by centrifugation and subsequently lysed in lysis buffer 1 (50 mM Tris-HCl, 150 mM NaCl, 1 mM EDTA, 1 mM dithiothreitol (DTT), 1 mM lysozyme, and 0.5% (v/v) Triton X-100, pH = 8.0). After centrifugation, the lysate supernatant was collected and loaded onto a GST column (GE Healthcare, USA) with an AKTA Explorer System (GE Healthcare, USA). After washing the

column-bound glutathione S-transferase (GST)-RANKL fusion protein, the RANKL was cleaved and eluted from the beads by on-column treatment with PreScission Protease (GE Healthcare, USA) for 4 hours at 4℃ (Figure S2A).

BL21 (DE3) containing the pET28a (+)-RANK plasmid was cultured in LB medium supplemented with 100 μg/ml kanamycin at 37 °C with agitation at 250 rpm until the $OD_{600}$ reached 0.6~0.8. Then 1 mM IPTG was added and the culture was incubated for 4 h. Following incubation, BL21 cells were harvested via centrifugation, and the precipitate which contained the inclusion bodies was re-suspended in 100 ml of ice-cold washing buffer 2 (50 mM Tris-HCl, 150 mM NaCl, 5 mM EDTA, 1% v/v Triton X-100, pH = 8.0). Finally, the cells were disrupted and homogenized using sonication, followed by thorough washing of the inclusion bodies.

To refold the RANK protein, the inclusion bodies firstly were dissolved in buffer 3 (6 M Guanidine–HCl, 50 mM Tris, 1 mM EDTA, 150 mM NaCl, 0.5 M L-arginine, 20% v/v glycerol, 10 mM DTT, pH = 8.0) at RT. After centrifugation, supernatant was collected and transferred into a dialysis bag and subsequently dialyzed by buffer 4 (2 M Guanidine–HCl, 0.5 M L-arginine, 50 mM Tris, 1 mM EDTA, 150 mM NaCl and 10% v/v glycerol, pH = 8.0) for 12 h. Then, a second dialysis was performed using buffer 5 (0.5 M Guanidine–HCl, 0.2 M L-arginine, 50 mM Tris, 1 mM EDTA, 150 mM NaCl and 5% v/v glycerol, pH = 8.0) for 12 h. Finally, the solution was dialyzed by PBS (containing 1 mM DDT, pH = 7.3). After that, the solution in the dialysis bag which containing RANK refolding protein was centrifuged and collected for further experiment (Figure S2B).

*1.2 Cellular thermal shift assay (CESTA)*

RAW264.7 cells were treated with IMB-R38 (10 μM) for 6 h and then washed three times with ice-cold PBS. The cell pellets were equally aliquoted into eight PCR tubes (50 μL/tube) and subjected to thermal challenge in a PCR thermocycler (AL050123, Bio-Rad, USA) under the following conditions: incubation at specified temperatures (range: 37-48.9°C) for 3 min, followed by equilibration at 25 °C for 3 min. To ensure complete cell lysis, samples underwent three freeze-thaw cycles alternating between liquid nitrogen immersion and 37 °C water bath. After centrifugation at 13,500 × g for 30 min at 4 °C, 40 μL of supernatant was mixed with

10 μL 5× loading buffer (P1040, Solarbio, Beijing, China,) and denatured at 100 °C for 10 min. The relative abundance of RANK and RANKL was measured by Western blot.

To further assess the concentration-dependent stabilizing effects of IMB-R38 on RANK and RANKL protein, RAW264.7 cells were pretreated with serially diluted IMB-R38 (0.1-10 μM) or equivalent DMSO for 6 h. The cell lysates were then subjected to thermal stress through sequential incubation at 45 °C for 3 min followed by 25 °C for 3 min. After high-speed centrifugation (13,500 × g, 30 min, 4 °C), the supernatant fractions were analyzed by Western blot.

**Supplementary Table S1.** Antibodies used in Western blot.

| Targetantigen | Vendor orSource | Catalog # | Workingconcentration | Persistent ID / URL |
|---|---|---|---|---|
| OPG | abcam | ab183910 | 1:500 | https://www.abcam.cn/products/primary-antibodies/osteoprotegerin-antibody-ab183910.html |
| BMP2 | abcam | ab284387 | 1:1000 | https://www.abcam.cn/products/primary-antibodies/bmp2-antibody-epr24209-61-ab284387.html |
| RUNX2 | abcam | ab236639 | 1:1000 | ab236639https://www.abcam.cn/products/primary-antibodies/runx2-antibody-epr22858-106-chip-grade-ab236639.html |
| β-catenin | abcam | ab32572 | 1:5000 | https://www.abcam.cn/products/primary-antibodies/beta-catenin-antibody-e247-chip-grade-ab32572 |
| Phospho-Smad1/5/9 | selleck | F2875 | 1:1000 | https://www.selleck.cn/antibodies/phospho-smad1-5-9-s463-465-467-antibody-k12g16.html |
| RANK | abcam | ab305233 | 1:1000 | https://www.abcam.cn/products/primary-antibodies/rank-antibody-epr26196-15-ab305233.html |
| RANKL | abcam | ab62516 | 1:1000 | https://www.abcam.cn/products/primary-antibodies/srankl-antibody-ab62516.html |
| MMP9 | abcam | ab38898 | 1:1000 | https://www.abcam.cn/products/primary-antibodies/mmp9-antibody-ab38898.html |
| NFATc1 | abcam | ab2976 | 1:1000 | https://www.abcam.cn/products/primary-antibodies/nfat2-antibody-7a6-ab2796.html |
| PI3K | CST | 4257 | 1:1000 | https://www.cellsignal.cn/products/primary-antibodies/pi3-kinase-p85-19h8-rabbit-monoclonal-antibody/4257 |
| p-PI3K | CST | 4228 | 1:1000 | https://www.cellsignal.cn/products/primary-antibodies/phospho-pi3-kinase-p85-tyr458-p55-tyr199-antibody/4228 |
| AKT | CST | 4691 | 1:1000 | https://www.cellsignal.cn/products/primary-antibodies/akt-pan-c67e7-rabbit-monoclonal-antibody/4691 |
| P-AKT | CST | 4060 | 1:2000 | https://www.cellsignal.cn/products/primary-antibodies/phospho-akt-ser473-d9e-rabbit-monoclonal-antibody/4060 |
| ERK | CST | 4695 | 1:1000 | https://www.cellsignal.cn/products/primary-antibodies/p44-42-mapk-erk1-2-137f5-rabbit-monoclonal-antibody/4695 |
| P-ERK | Biodragon | BD-PB5113 | 1:1000 | https://www.biodragon.cn/cn/goods/goodsView?GoodsId=79598&Catalog= |
| JNK | Biodragon | RM1210 | 1:1000 | https://www.biodragon.cn/cn/goods/goodsView?GoodsId=53634&Cat |

| Targetantigen | Vendor orSource | Catalog # | Workingconcentration | Persistent ID / URL |
|---|---|---|---|---|
| | | | | alog= |
| p-JNK | CST | 4668 | 1:1000 | https://www.cellsignal.cn/products/primary-antibodies/phospho-sapk-jnk-thr183-tyr185-81e11-rabbit-monoclonal-antibody/4668 |
| P38 | Bioworld | AP0424 | 1:1000 | https://www.bioworlde.com/Primary-Antibodies/139978.html |
| p-P38 | abcam | ab195049 | 1:1000 | https://www.abcam.cn/products/primary-antibodies/p38-phospho-t180-y182-antibody-epr18120-ab195049 |
| P65 | abcam | Ab16502 | 1：1000 | https://www.abcam.cn/products/primary-antibodies/nf-kb-p65-antibody-ab16502 |
| P-P65 | Bioss | bs-0982R | 1:1000 | https://www.biosschina.com/productDetail?goods_id=7439 |
| lkB | Biodragon | RM0145 | 1:1000 | https://www.biodragon.cn/cn/goods/goodsView?GoodsId=52581&Catalog= |
| p-lkB | abcam | 9246 | 1:1000 | https://www.cellsignal.cn/products/primary-antibodies/phospho-ikappab-alpha-ser32-36-5a5-mouse-monoclonal-antibody/9246 |
| α-tubulin | Proteintech | 66031-1-Ig | 1:50000 | https://www.ptgcn.com/products/tubulin-Alpha-Antibody-66031-1-Ig.htm |
| GAPDH | Proteintech | 10494–1-AP | 1:50000 | https://ptgcn.com/products/GAPDH-Antibody-10494-1-AP.htm |

**Supplementary Table S2.** Primers Sequences of the RT-qPCR.

| Gene | Forward Primer | Reverse Primer |
|---|---|---|
| *Bmp2b* | GAAGTATCCGAGGAGGCTGA | CCTCCACCACCATGTCCT |
| *Runx2a* | AGACTCCGACCTCACGACAACC | GGCAGCACCGAGCACAGAAAG |
| *Runx2b* | GAAGCGGAAGAGGGAAGAGC | AATGGCTCTGTGGTAAGTGGC |
| *Sp7* | AAGAAACCTGTCCACAGCTG | GAGGCTTTACCGTACACCTT |
| *Alp* | CTGGTGGAAGGTGGACGCATTG | CGTGTCATACTCGCTTGTCAGGAG |
| *Oc* | TGGCCTCTATCATCATGAGACAGA | CTCTCGAGCTGAAATGGAGTCA |
| *Mmp9* | CAGAGAGGAAAAGGCAAGGTG | AGGAATACATCATGTGAATCAATG |
| *Mmp2* | CTGGGAACAAGTTCTGGAGATAC | GTGAGTCTTTGAAGAAGTAGCTG |
| *Mmp13* | AGACCAGGACACACTCGCAGAG | CTGCTGCATCTCCTTGAGCCT |
| *Il-6* | GCGTCCTGACGTGGTATAAAG | GTCGTTTGGTGCTGTGTTTG |
| *Aggrecan* | ACAGGTGTGGTCTTCCATTAC | GACTGCGCCGATACTCTTAC |

A

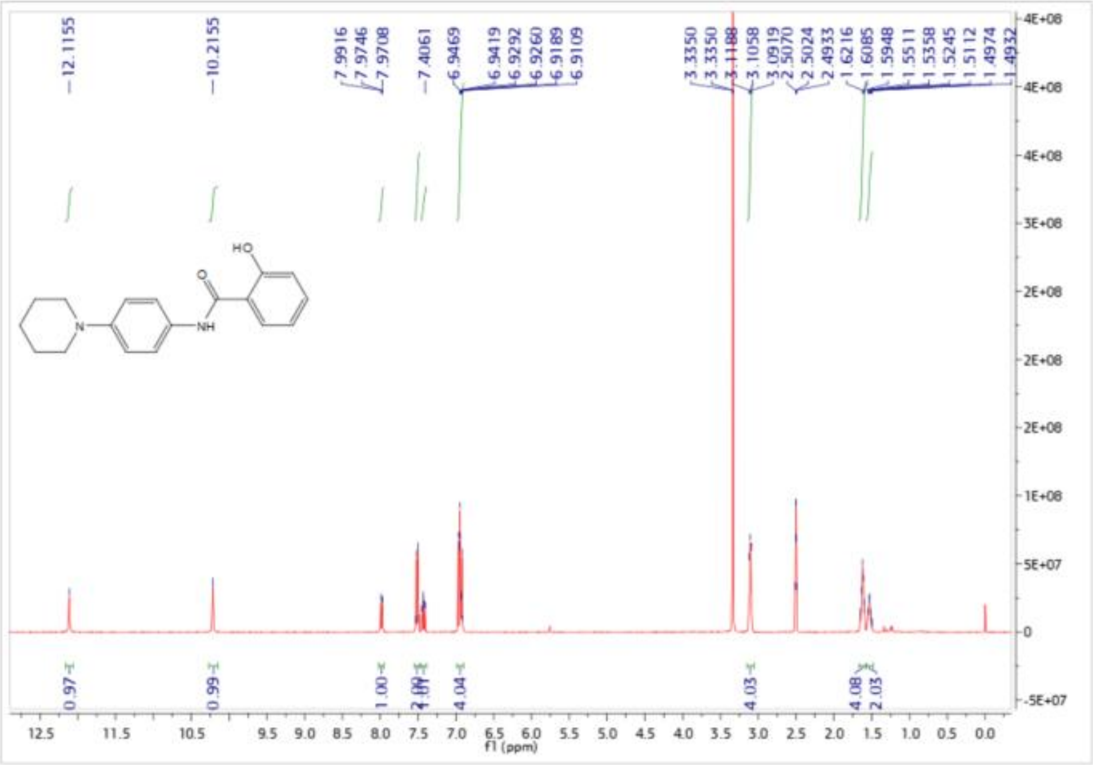


B

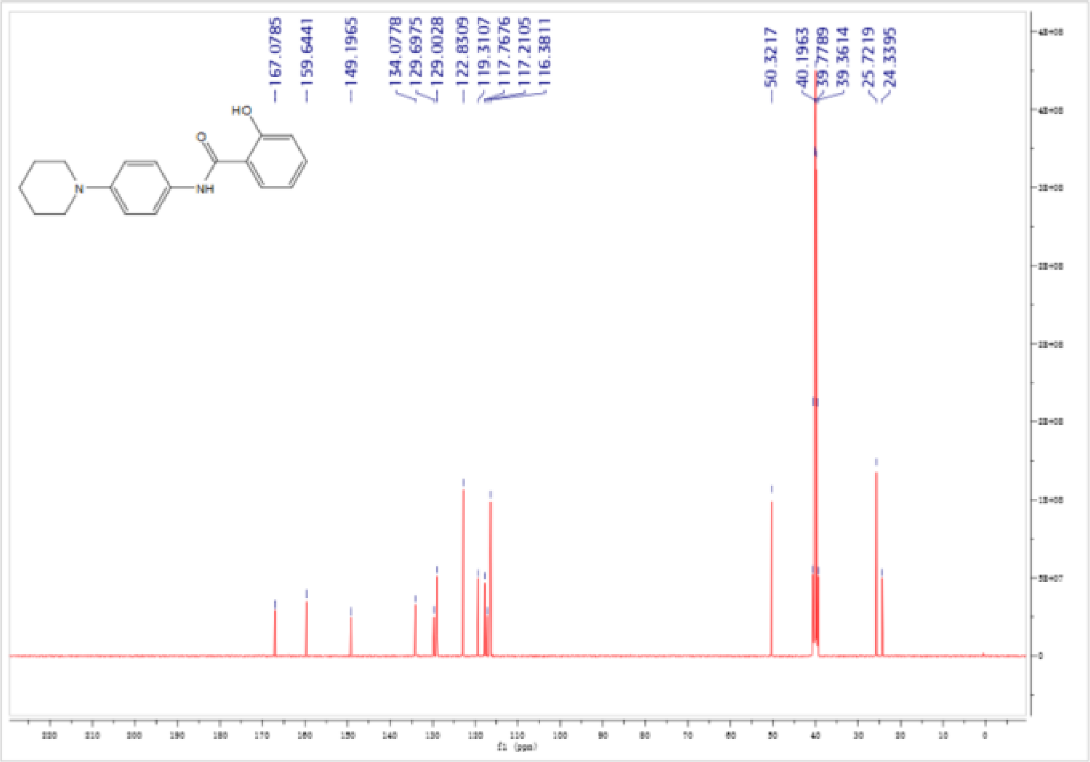


**Supplementary Figure S1.** (A, B) $^{1}H$ NMR, $^{13}C$ NMR for compound IMB-R38.

**A**

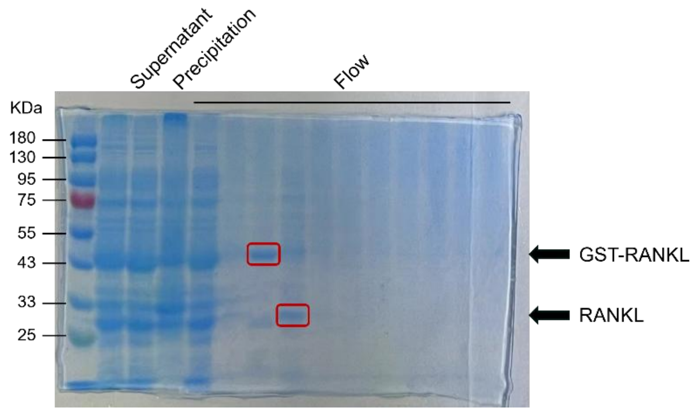


**B**

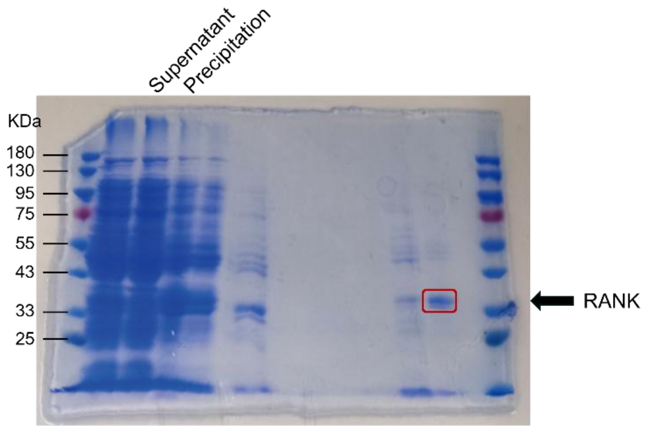


**C**

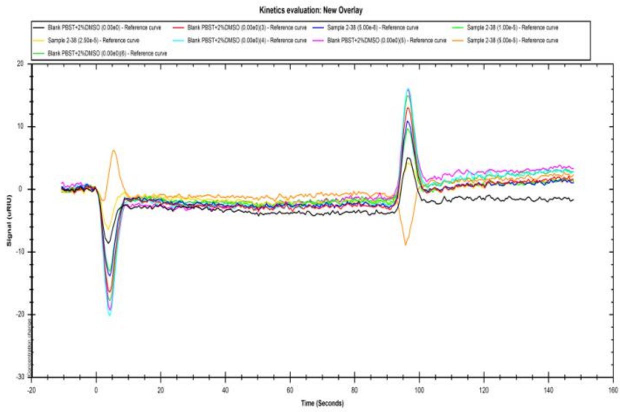


**Supplementary Figure S2.** (A-B) The SDS-PAGE of the recombinant GST-tagged RANKL and his-tagged RANK. The red boxes are labeled with purified RANKL or RANK. (C) SPR sensorgram for the interaction between IMB-R38 and RANKL protein.

## Supplementary Figure S3. All uncropped membranes

Figure 1B

Cyc-RANK

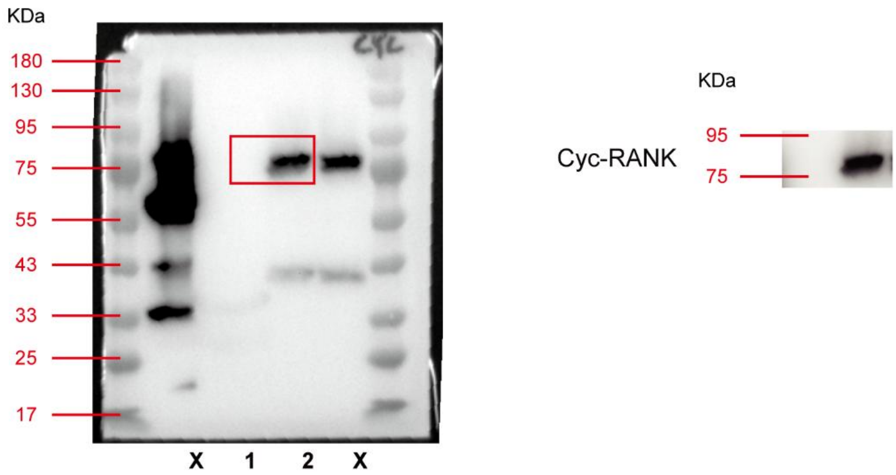


1: pAD-T/pBD-Lam

2: pAD-RANKL/pBD-RANK

HA-RANKL

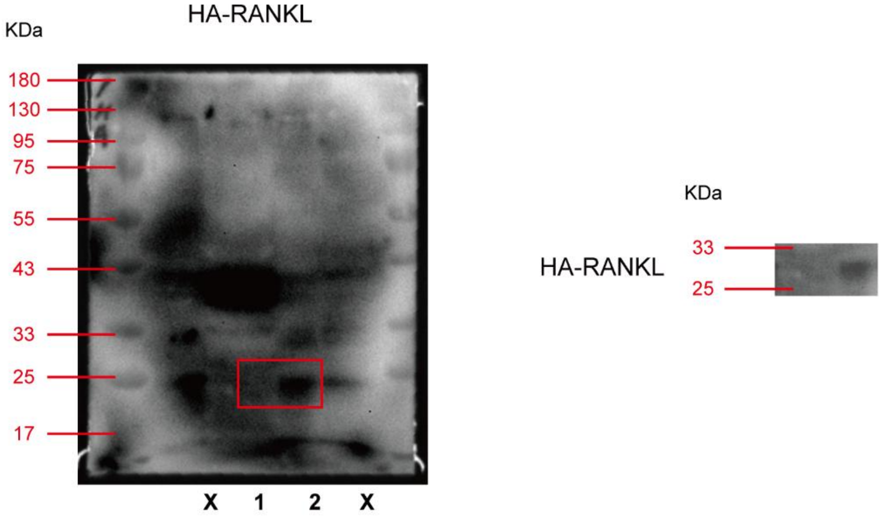


1: pAD-T/pBD-Lam

2: pAD-RANKL/pBD-RANK

Figure 2E

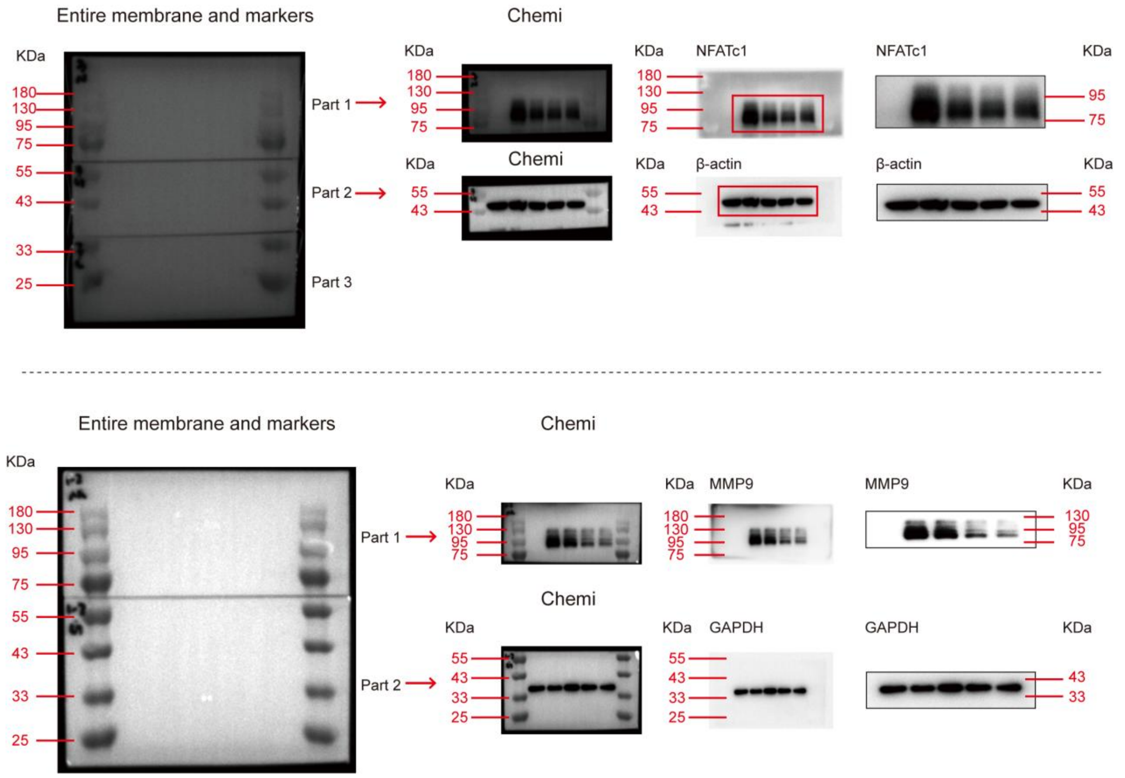

Entire membrane and markers
Chemi
KDa
180
130
95
75
55
43
33
25
Part 1
Part 2
Part 3
NFATc1
NFATc1
β-actin
β-actin
Entire membrane and markers
Chemi
Part 1
Part 2
MMP9
MMP9
GAPDH
GAPDH

Figure 3C

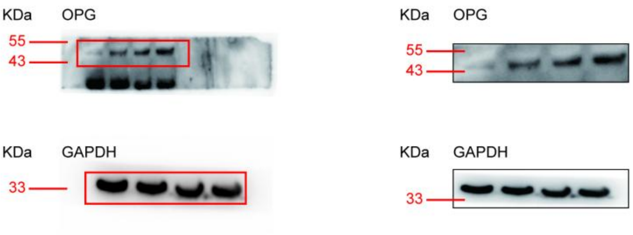


Figure 3F

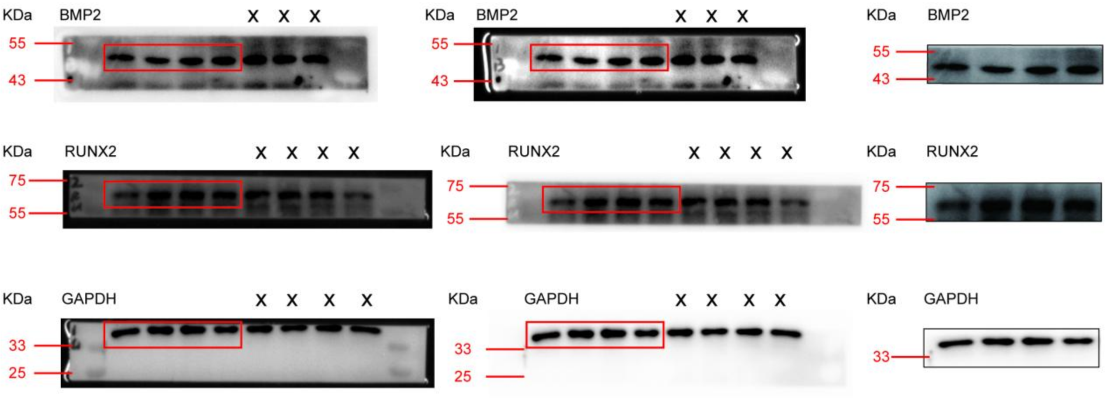


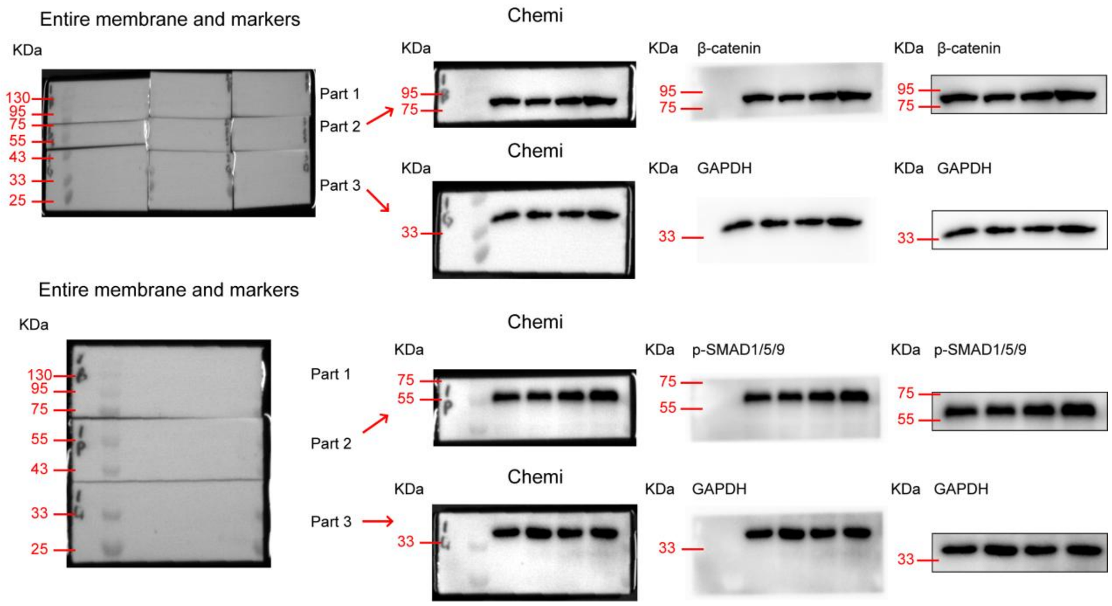

Figure 5A

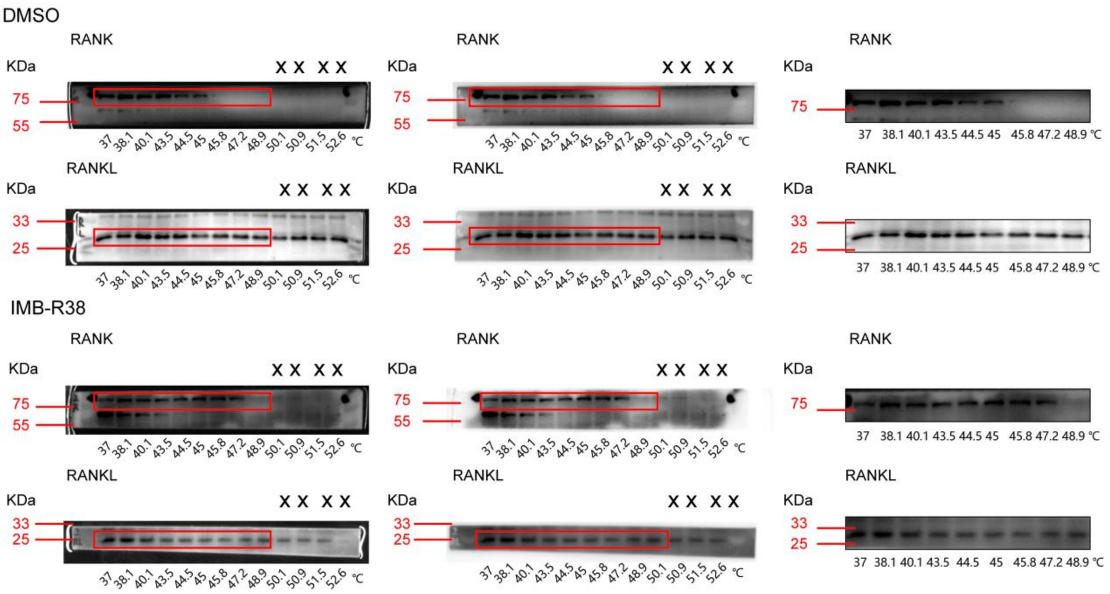


Figure 5D

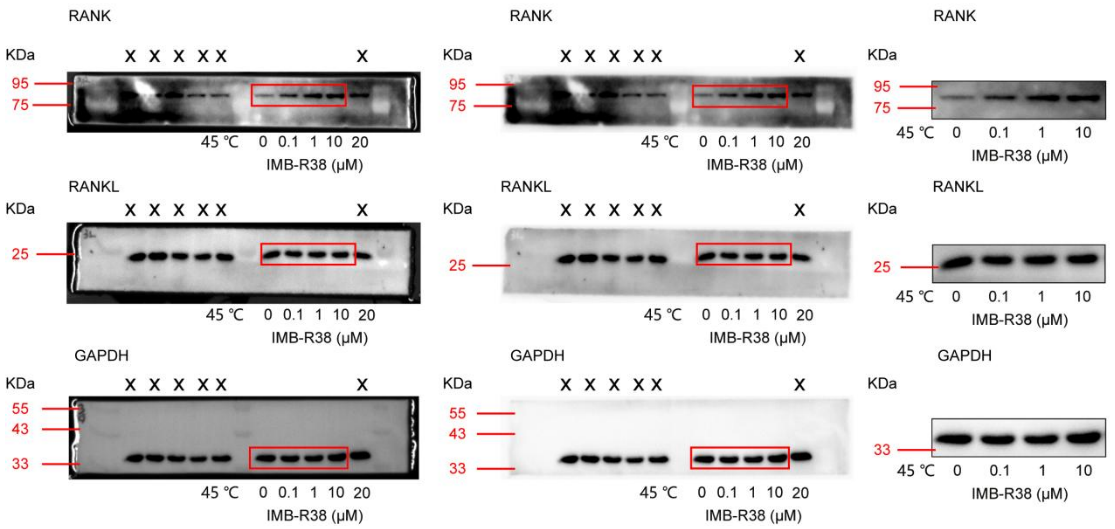

Figure 6A

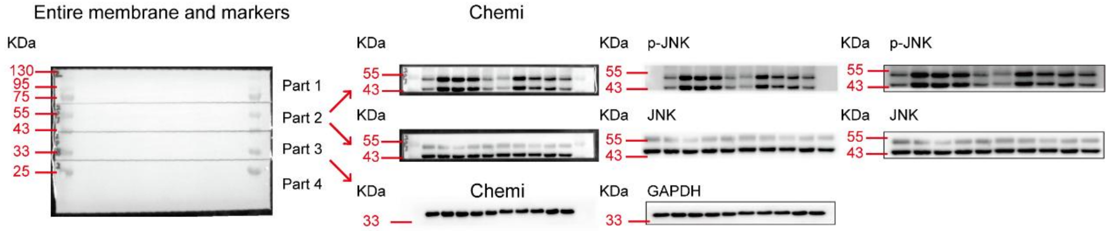


Figure 6C

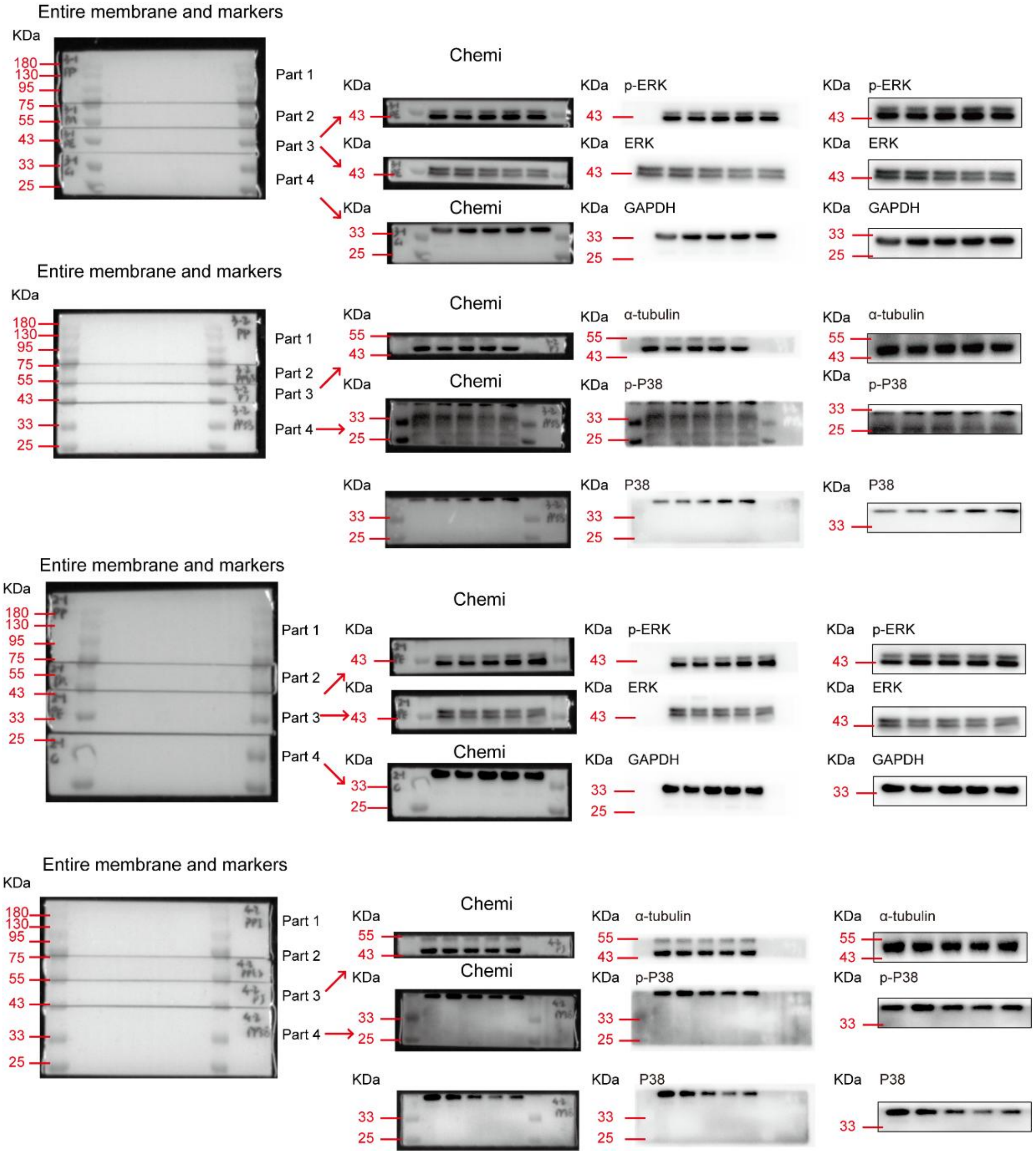

Figure 6D

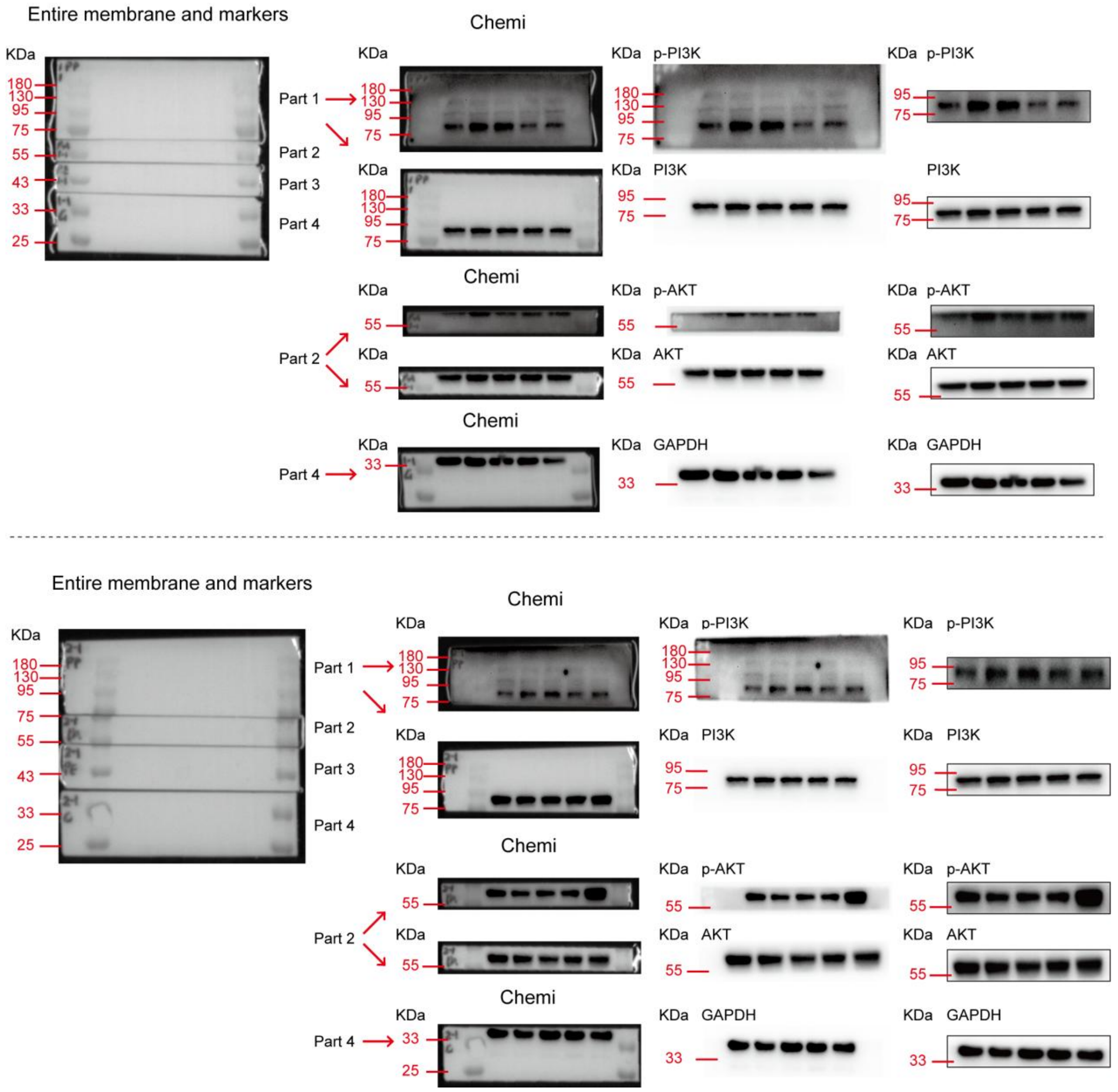
Entire membrane and markers
Chemi
KDa
p-PI3K
PI3K
p-AKT
AKT
GAPDH
Part 1
Part 2
Part 3
Part 4
180
130
95
75
55
43
33
25

Figure 6E

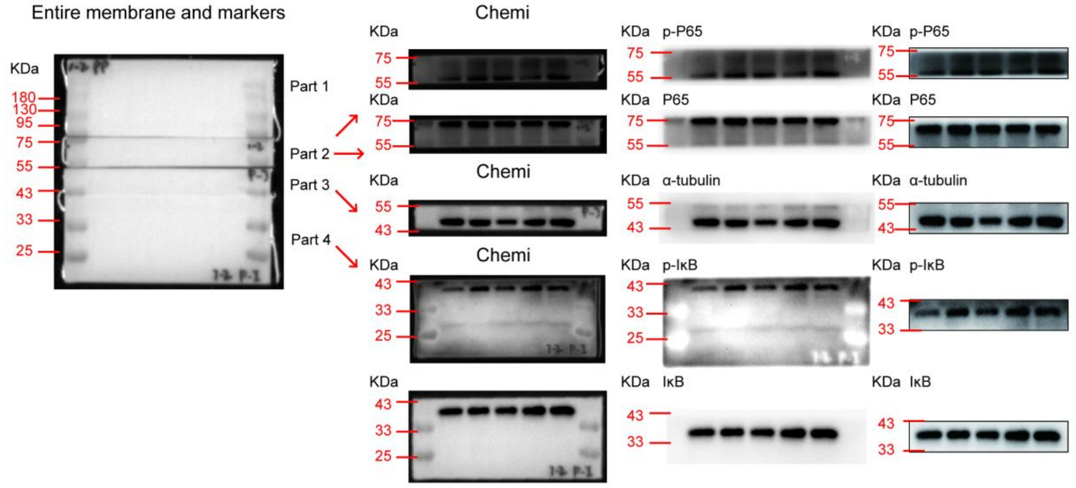

Entire membrane and markers
KDa
180
130
95
75
55
43
33
25
Part 1
Part 2
Part 3
Part 4
Chemi
p-P65
P65
α-tubulin
p-IκB
IκB


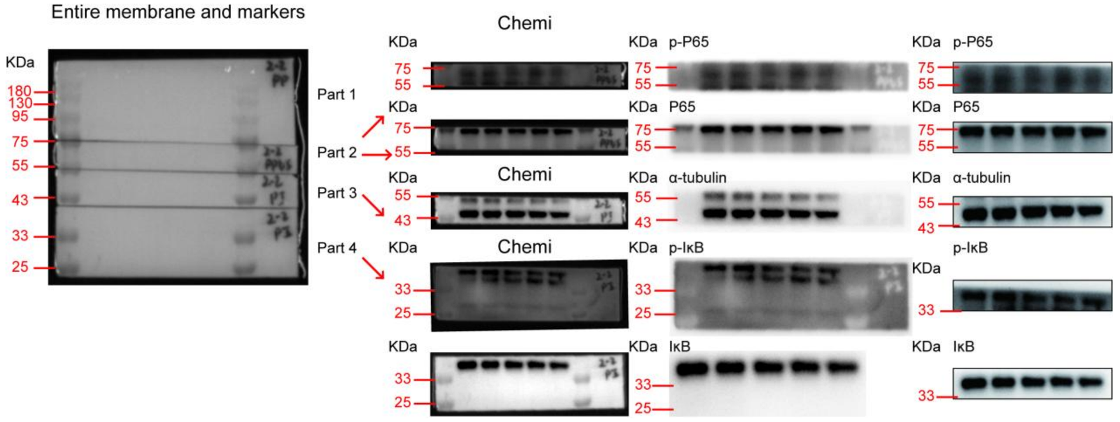

Entire membrane and markers
KDa
180
130
95
75
55
43
33
25
Part 1
Part 2
Part 3
Part 4
Chemi
p-P65
P65
α-tubulin
p-IκB
IκB
